# 20-Hydroxyeicosatetraenoic Acid Contributes to the Inhibition of K+ Channel Activity and Vasoconstrictor Response to Angiotensin II in Rat Renal Microvessels

**DOI:** 10.1371/journal.pone.0082482

**Published:** 2013-12-04

**Authors:** Fan Fan, Cheng-Wen Sun, Kristopher G. Maier, Jan M. Williams, Malikarjuna R. Pabbidi, Sean P. Didion, John R. Falck, Jialong Zhuo, Richard J. Roman

**Affiliations:** 1 Department of Pharmacology and Toxicology, University of Mississippi Medical Center, Jackson, Mississippi, United States of America; 2 Department of Pharmaceutical Sciences, North Dakota State University, Fargo, North Dakota, United States of America; 3 Division of Vascular Surgery and Endovascular Services, SUNY Upstate Medical University, Syracuse, New York, United States of America; 4 Department of Molecular Genetics, University of Texas Southwestern Medical Center, Dallas, Texas, United States of America; INSERM, France

## Abstract

The present study examined whether 20-hydroxyeicosatetraenoic acid (HETE) contributes to the vasoconstrictor effect of angiotensin II (ANG II) in renal microvessels by preventing activation of the large conductance Ca^2+^-activated K^+^ channel (K_Ca_) in vascular smooth muscle (VSM) cells. ANG II increased the production of 20-HETE in rat renal microvessels. This response was attenuated by the 20-HETE synthesis inhibitors, 17-ODYA and HET0016, a phospholipase A_2_ inhibitor AACOF_3_, and the AT_1_ receptor blocker, Losartan, but not by the AT_2_ receptor blocker, PD123319. ANG II (10^-11^ to 10^-6^ M) dose-dependently decreased the diameter of renal microvessels by 41 ± 5%. This effect was blocked by 17-ODYA. ANG II (10^-7^ M) did not alter K_Ca_ channel activity recorded from cell-attached patches on renal VSM cells under control conditions. However, it did reduce the NPo of the K_Ca_ channel by 93.4 ± 3.1% after the channels were activated by increasing intracellular calcium levels with ionomycin. The inhibitory effect of ANG II on K_Ca_ channel activity in the presence of ionomycin was attenuated by 17-ODYA, AACOF_3_, and the phospholipase C (PLC) inhibitor U-73122. ANG II induced a peak followed by a steady-state increase in intracellular calcium concentration in renal VSM cells. 17-ODYA (10^-5^ M) had no effect on the peak response, but it blocked the steady-state increase. These results indicate that ANG II stimulates the formation of 20-HETE in rat renal microvessels via the AT_1_ receptor activation and that 20-HETE contributes to the vasoconstrictor response to ANG II by blocking activation of K_Ca_ channel and facilitating calcium entry.

## Introduction

 Angiotensin II (ANG II) plays a crucial role in the regulation of body fluid volume homeostasis and the long term control of arterial pressure by altering sodium excretion and vascular tone. ANG II is a potent constrictor of renal microvessels that regulates renal blood flow and glomerular filtration rate [1-3]. However, the underlying mechanism is not completely understood. Previous studies have demonstrated that ANG II activates phospholipase A_2_ (PLA_2_) and phospholipase C (PLC) in aortic VSM cells to increase the release of arachidonic acid (AA) and the production of prostaglandin E_2_, prostacyclin, EETs and 12-, 19- and 20-hydroxyeicosatetraenoic acid (HETE) [4-6]. Several of these metabolites modulate the vasoconstrictor response to ANG II [1,4,7]. For example, the renal vasoconstrictor response to ANG II is potentiated by blockade of cyclooxygenase and the ANG II-induced increase in intracellular calcium concentration ([Ca^2+^]_i_) in cultured renal VSM cells is attenuated by lipoxygenase inhibitors [6,8]. Our lab has also reported that the renal vasoconstrictor and pressor responses to ANG II in rats are attenuated by blockade of the formation of 20-HETE [1]. However, the mechanism by which 20-HETE contributes to the vasoconstrictor response to ANG II remains to be determined. The present study examined the effects of ANG II on the formation of 20-HETE, vascular tone, K_Ca_ channel activity and intracellular calcium concentration in renal microvessels in the presence and absence of inhibitors of the synthesis of 20-HETE.

## Materials and Methods

### Animals

 Experiments were performed on 178 male, 12-14 week-old SD rats purchased from Charles River Laboratories (Wilmington, MA). The rats were housed in the animal care facilities at the Medical College of Wisconsin and the University of Mississippi Medical Center that are both approved by the American Association for the Accreditation of Laboratory Animal Care. The rats had free access to food and water through the study and all protocols involving animals received prior approval by the Institutional Animal Care and Use Committees (IACUC) of the Medical College of Wisconsin and the University of Mississippi Medical Center.

### Measurement of 20-HETE production in renal microvessels

 Rat renal microvessels were isolated using an Evans blue sieving procedure similar to that previously described in the cerebral circulation [9]. The rats were anesthetized with isoflurane and a cannula was placed in the lower aorta below the renal arteries. The aorta above the renal arteries was tied off and the kidneys were flushed with 10 ml of iced-cold low calcium Tyrode’s solution containing (in mM): 145 NaCl, 5 KCl, 4.2 NaHCO_3,_ 1 MgCl_2,_ 0.05 CaCl_2_, 10 HEPES, and 10 glucose. Then, 5 ml of the Tyrode’s solution containing 3% albumin stained with 1% Evans blue was injected to fill the renal microcirculation. The kidney was rapidly removed and hemisected, and the inner medulla and outer medulla were excised. Pieces of the renal cortex were forced through a 150-μm stainless steel sieve with the barrel of a 30 ml glass syringe to mechanically separate tubules and glomeruli from the vascular trees. The tissue retained on the screen was repeatedly rinsed with ice-cold physiological salt solution (PSS) containing (in mM): 119 NaCl, 4.7 KCl, 1.2 MgSO_4_, 1.6 CaCl_2,_ 1.2 NaH_2_PO_4_, 18 NaHCO_3_, 0.03 EDTA, 10 glucose, and 5 HEPES. The retained vascular tissue on the top of the screen was collected, resuspended in ice-cold PSS solution, and any adherent tubules were removed from the vessels by microdissection using a stereomicroscope. 

 The freshly isolated renal microvessels were incubated in 1 ml of PSS containing: a) vehicle, b) ANG II alone (10^-7^ M), c) ANG II plus 17-ODYA (10^-5^ M), d) ANG II plus HET0016 (10^-8^ M), e) ANG II plus Losartan (10^-6^ M), f) ANG II plus AACOF_3_ (2 X 10^-5^ M), and g) ANG II plus PD123319 (10^-7^ M) in the presence of 2 µM indomethacin, 1 mM NADPH and 40 μM of AA at 37° C for 30 min. Stock solution of ANG II (Sigma A9525) was dissolved in distilled water and 0.05% acetic acid at a concentration of 10^-4^ M. Stock solutions of HET0016 (Enzo Scientific) and indomethacin (Sigma I7378), 17-ODYA (Enzo BML, EI-1030), U73122 Sigma U6756), AACOF3 and AA were prepared in ethanol at concentrations of 10^-2^ M to 10^-3^ M. Losartan Potassium (Sigma 61188) was dissolved in assay buffer at a concentration of 10^-3^ M. All of the drugs were used in 1:500 to 1:1000 dilutions in PSS or assay buffer to the final concentrations used in the various experiments. 

We have previously reported that the formation of 20-HETE by the CYP4A enzymes is linearly related to PO_2_ over the range of 20-140 torr in the incubation media[10]. Thus, the renal microvessels were shaken under an atmosphere of 100% oxygen, which we have previously reported is necessary to maintain a PO_2_ of approximately 100 torr [11] in the incubation media [12,13]. The reactions were stopped by acidification to pH 3.5 with formic acid and the vessels were homogenized with a ground glass homogenizer on ice until no tissue was visible. A 100 µl aliquot was taken for measurement of protein concentration and the remainder of the sample was extracted twice with 3 ml of ethyl acetate after the addition of 2 ng of an internal standard d6-20-HETE. After centrifugation, the organic phase was dried under nitrogen and stored at -80° C until further analysis.

 The metabolites of AA were separated using a Dionex (Sunnyvale, CA) HPLC system and an ABsciex 4000 Q trap tandem mass spectrometer with electrospray ionization (ABsciex, Foster, City, CA.). Separation of the metabolites was achieved using a reverse phase column (Beta basic C18, 150 X2.1 mm, 3 μm; Thermo Hypersil-Keystone, Bellefonte, PA) and the following mobile phase conditions at a flow rate of 300 µl/minute. The column was first equilibrated with 66.7% of a mobile phase A containing water/acetonitrile/methanol/acetic acid 90/8.5/1.4/0.1 (v/v/v/v), and 33.3% mobile phase B, acetonitrile/methanol 85/15 for 5 minutes following injection of the sample. The percentage of mobile phase B was ramped to 53.5% over a 10 min period and then held there for 5 minutes followed by a linear increase to 94.4% mobile phase B over a 7 min period and then held there for another 5 min. Column temperature was maintained at 35° C.

 The products were ionized using the negative ion mode and analyzed using multiple reaction monitoring (MRM) with the following instrument settings: Electrospray voltage -4500 volts, curtain gas 30, gas 1-50, temperature 600, gas 2-50, and unit resolution. The following transitions were monitored for each metabolite of AA; m/z 337-207 (14,15-DIHETE), m/z 337-167 (11,12-DIHETE), m/z 337-127 (8,9-DIHETE), m/z 319-231 (19-HETE), m/z 319-245 (20-HETE), m/z 319-261 (18-HETE), m/z 337-145 (5,6-DIHETE), m/z 319-233 (16-HETE), m/z 319-175 (15-HETE), m/z 319-149 (11-HETE), m/z 319-179 (12-HETE), m/z 319-155 (8HETE), m/z 319-203 (5-HETE), m/z 319-175, (14,15-EET), m/z 319- 167 (11,12-EET), m/z 319-127 (8,9-EET), m/z 319-191 (5,6-EET), and m/z 325-281/307 (d^6^ 20-HETE) for the internal standard. Standard curves were generated over a range of 0.02 to 20 ng for each metabolite. 

### Expression of ANG II receptors in renal microvessels

 Microdissected renal microvessels were placed into ice cold RNAlater solution (Life Technologies, Grand Island, NY) overnight. They were homogenized in TRIzol solution (Life Technologies, Grand Island, NY) using a FastPrep-24 homogenizer (MP Biomedicals, Santa Ana, CA), and RNA was extracted according to manufacturer’s instructions. Aliquots of the RNA (1 μg) were added to a 20 μl reverse transcription reaction using the iScript cDNA Synthesis Kit (Bio-Rad, Hercules, CA). The reactions were incubated at 25° C for 5 min, 42° C for 30 min followed by inactivation at 85° C for 5 min. The 25 μl PCR reactions contained 25 ng of the forward and reverse primers, 20 mM Tris-HCl buffer (pH 8.4), 50 mM KCl, 1.5 mM MgCl_2_, 200 μM of each dNTP, 0.5U Taq DNA Polymerase (QIAGEN, Valencia, CA). The reaction mixtures were initially denatured at 94° C for 5 min and then cycled 35 times at 94° C (denaturation) for 30 sec, 64° C (annealing) for 30 sec, and 72° C (elongation) for 30 sec followed by extension for 7 min at 72° C. The RT-PCR products were separated on 1 % agarose gel in a 1X Tris-borate-EDTA (TBE) buffer containing ethidium bromide (Sigma, St. Louis, MO) and the band intensity analyzed using a ChemiDoc MP Imaging System (Bio-Rad, Hercules, CA). The primer sequences for rat AT_1A_R (NM_030985) corresponded to 5’-CGT CAT CCA TGA CTG TAA AAT TTC-3’ (sense, 1071-1094) and 5’-GGG CAT TAC ATT GCC AGT GTG-3’ (antisense, 1376-1356). The final PCR product size is 306 bp. The primer sequences for rat AT1BR (NM_031009) corresponded to 5’-CAT TAT CCG TGA CTG TGA AAT TG-3’ (sense, 972-994) and 5’-GCT GCT TAG CCC AAA TGG TCC-3’ (antisense: 1316-1296) [14]. The final PCR product size is 345 bp. The primer sequences for rat AT_2_R (NM_012494) corresponded to 5’-GCT GTG GCT GAC TTA CTC CT-3’ (sense, 259-278) and 5’-GGT CAC GGG TAA TTC TGT TC-3’ (antisense, 757-738) [15], the final PCR product size is 499 bp. The primer sequences for rat GAPDH (NM_017008) corresponded to 5’- CCC CTT CAT TGA CCT CAA CTA C-3’ (sense, 174-195) and 5’-ATG CAT TGC TGA CAA TCT TGA G-3’ (antisense, 520-499), the final PCR product size is 347 bp. The primer sequences for rat von Willebrand factor (vWf) (NM_053889) corresponded to 5’- CTC CCA GCA CTA ACT GCA CCA GC-3’ (sense, 843-865) and 5’- CAA GAA CAG TCA GAG CTC TGC AC-3’ (antisense, bp 1278-1256), the final PCR product size is 436 bp. 

### Western blotting to verify the purity of isolated renal microvessel preparations

The renal microvessels were powdered in liquid nitrogen and then homogenized in an ice cold RIPA buffer (R0278, Sigma-Aldrich, St. Louis, MO) using a ground glass homogenizer followed by the FastPrep-24 homogenizer (MP Biomedicals, Santa Ana, CA). The samples (50 µg) were separated by electrophoresis on 10% SDS-polyacrylamide gel, transferred to nitrocellulose membranes using Trans-Blot Turbo Transfer System (Bio-Rad, Hercules, CA) and the membranes were blocked at room temperature for one hour in a buffer containing 10% nonfat dry milk. The membranes were incubated overnight at 4° C with a 1:500 dilution of anti-alkaline phosphatase primary antibody (sc-137213, Santa Cruz Biotechnology, Santa Cruz, CA) followed by a 1:5000 dilution of a horseradish peroxidase coupled anti-mouse secondary antibody (sc-2005, Santa Cruz Biotechnology, Santa Cruz, CA) for 1 h. The membrane was re-probed with 1: 8000 dilution of anti-beta actin (ab6276, Abcam, Cambridge, MA) and 1: 20000 of anti-mouse antibody. The blots were exposed to SuperSignal West Dura Extended Duration Substrate (34076, Thermo Scientific, Pittsburgh, PA) and the relative intensities of the bands at 80 KD for alkaline phosphatase and 42 KD for beta actin were imaged using a ChemiDoc photodocumentation system (Bio-Rad, Hercules, CA) .

### Vascular reactivity studies

 Rats were anesthetized with pentobarbital (50 mg/kg body weight, i.p.) and the abdominal aorta was cannulated below the left renal artery. The kidneys were flushed via the aorta with 10 ml of ice-cold low calcium Tyrode’s solution. The left kidney was removed and renal microvessels (50 to 100 μm) were isolated by microdissection, mounted on glass micropipettes and intraluminal pressure was maintained at 80 mmHg. The vessels were de-endothelialized by perfusion with anti-factor VIII-related antigen antibody (1:1000, Sigma-Aldrich, St. Louis, MO) and complement (20 mg/ml, Sigma-Aldrich, St. Louis, MO) for 5 min as previously described to remove the influence of endothelial dependent vasodilatory factors (NO, prostaglandins and EETs) on the vascular response to ANG II [16]. After a 30-minute equilibration period, the effects of various agonists and inhibitors on the inner diameter of the vessel were determined using a video system composed of a stereomicroscope (Carl Zeiss Inc. Thornwood, NY), a television camera (KP-130 AV, Hitachi, Woodbury, NY), a videocassettes recorder (A6 to 7330, Panasonic) and a video caliper (VIA-100, Boeckeler Instrument Co.). The vessels were perfused with PSS that was equilibrated with a 95% O_2_, 5% CO_2_ gas mixture to maintain pH at 7.4. Indomethacin (5 x 10^-6^ M) and baicalein (5 x 10^-6^ M) were added to the bath to block the endogenous metabolism of AA via the cyclooxygenase and lipoxygenase pathways as previously described [17,18]. The vasoconstrictor response to ANG II was evaluated before and after the addition of vehicle or 17-ODYA (10^-5^ M) to the bath.

### Patch clamp studies

#### Isolation of renal VSM cells

The kidneys were flushed with ice-cold low calcium Tyrode’s solution and renal microvessels were microdissected. The vessels were then sequentially incubated in 1 ml of a low calcium Tyrode’s solution containing 1.5 mg/ml, papain (14 U/mg), 1 mg/ml DTT for 15 minutes at 37° C, followed by incubation in low calcium Tyrode’s solution containing 90 U/ml elastase, 10000 U/ml soybean trypsin inhibitor, and 196 U/ml collagenase for about 18 minutes or until free cells were found in the media. The supernatant was collected and the cells were centrifuged at 500g for 5 minutes, resuspended in fresh low calcium Tyrode’s solution, and stored at 4° C. Patch-clamp experiments were performed within 4 hours after cell isolation.

#### Current Recordings

K^+^ currents were recorded using the cell-attached patch-clamp technique at room temperature as we have described previously [18-20]. The patch clamp pipettes were constructed from 1.5 mm borosilicate glass pulled using a two-stage micropipette puller (Model PC-87, Sutter Instrument Co., San Rafael, CA) and heat-polished using a microforge. The pipettes had a tip resistance of 8-10 megohms and were back-filled with a solution containing (in mM): 145 KCl, 1.8 CaCl_2_, 1.1 MgCl_2_, 5 HEPES (pH 7.4). Renal VSM cells were allowed to settle and attach to a glass coverslip that formed the bottom of a 1 ml perfusion chamber mounted on the stage of an inverted microscope. After positioning the tip of a pipette on a cell, a high resistance seal (5-20 GW) was formed by applying a light suction. A List EPC-7 patch-clamp amplifier (List Biological Laboratories, Inc., Campbell, CA) was used to clamp pipette potential and record single-channel currents. The amplifier output signals were filtered at 2 kHz using an eight-pole Bessel filter (Frequency Devices Inc, Haverhill, MA). Single-channel current tracings were recorded from cell-attached membrane patches of renal VSM cells bathed in a high potassium solution containing (in mM): 145 KCl, 1.8 CaCl_2_, 1.1 MgCl_2_, 10 glucose, 5 HEPES (pH 7.4) to null the membrane potential or a normal physiological solution (PSS) containing (in mM): 140 NaCl, 5.4 KCl, 1 MgCl_2_, 1.8 CaCl_2_, 5 HEPES, 10 Glucose (pH 7.4). The currents were digitized at a rate of 10 kHz and stored on a computer hard disk for later analysis. Data acquisition and analysis was performed using pClamp software. The open-state probability (NP_o_) of the single-channel currents were expressed as a percentage of the total recording time in which the channel was open and was calculated using the following equation: 

${\text{NP}}_{\text{o}}{\text{ = ((ST}}_{\text{j}}\text{ X j)/T X 100}$

Where T_j_ is the sum of the open time at a given conductance level, j represents multiples of a given conductance, and T is the total recording time. 

### Effect of ANG II on intracellular calcium concentration

 Intracellular calcium concentration [Ca^2+^]_i_ was measured in freshly isolated renal VSM cells at room temperature using the calcium-sensitive dye Fura-2-acetoxymethyl aster (Fura-2 AM, Life Technologies, Grand Island, NY). After the VSM cells were isolated as described above, they were loaded with Fura-2 by incubation in low calcium, high potassium solution containing 4 x 10^-6^ M Fura-2 AM mixed, 2 x 10^-8^ M pluronic acid and 1 mg/ml albumin for 60 minutes in the dark at room temperature. The cells were then transferred into a 1 ml perfusion chamber on a heated platform to control bath temperature at 37° C. After the VSM cells attached to the bottom of the chamber, they were superfused with a PSS solution for 30 minutes at 37° C. Intracellular calcium concentrations were imaged using a 40X fluorescence objective on an inverted microscope. The VSM cells were excited alternately at 340- and 380- nm with a xenon illuminator. Fluorescence intensity was detected using a low-light level integrating CCD camera and processed with an InCyt Im2^TM^ Imaging System (Intracellular Imaging Inc., Cincinnati, OH). The [Ca^2+^]_i_ was calculated from the 340/380 nm fluorescence intensity ratio according to a standard curve generated with Fura 2 and calcium standard solutions with various known concentrations. The peak response was measured within the first 100 msec, while the steady state response was measured between 100-200 msec after the administration of ANG II.

### Statistics

Mean values ± SEM are presented. The significance of the differences in mean values between and within groups was determined using an analysis of variance for repeated measures followed by a Duncan’s multiple range test. An unpaired T-test was used to compare the differences in the production of metabolites of AA in control and ANG II treated microvessels. Single-channel conductances were fitted using least-squares linear regression. A P value < 0.05 was considered to be statistically significant.

## Results

### Expression of ANG II receptors in renal microvessels

**Figure 1:**
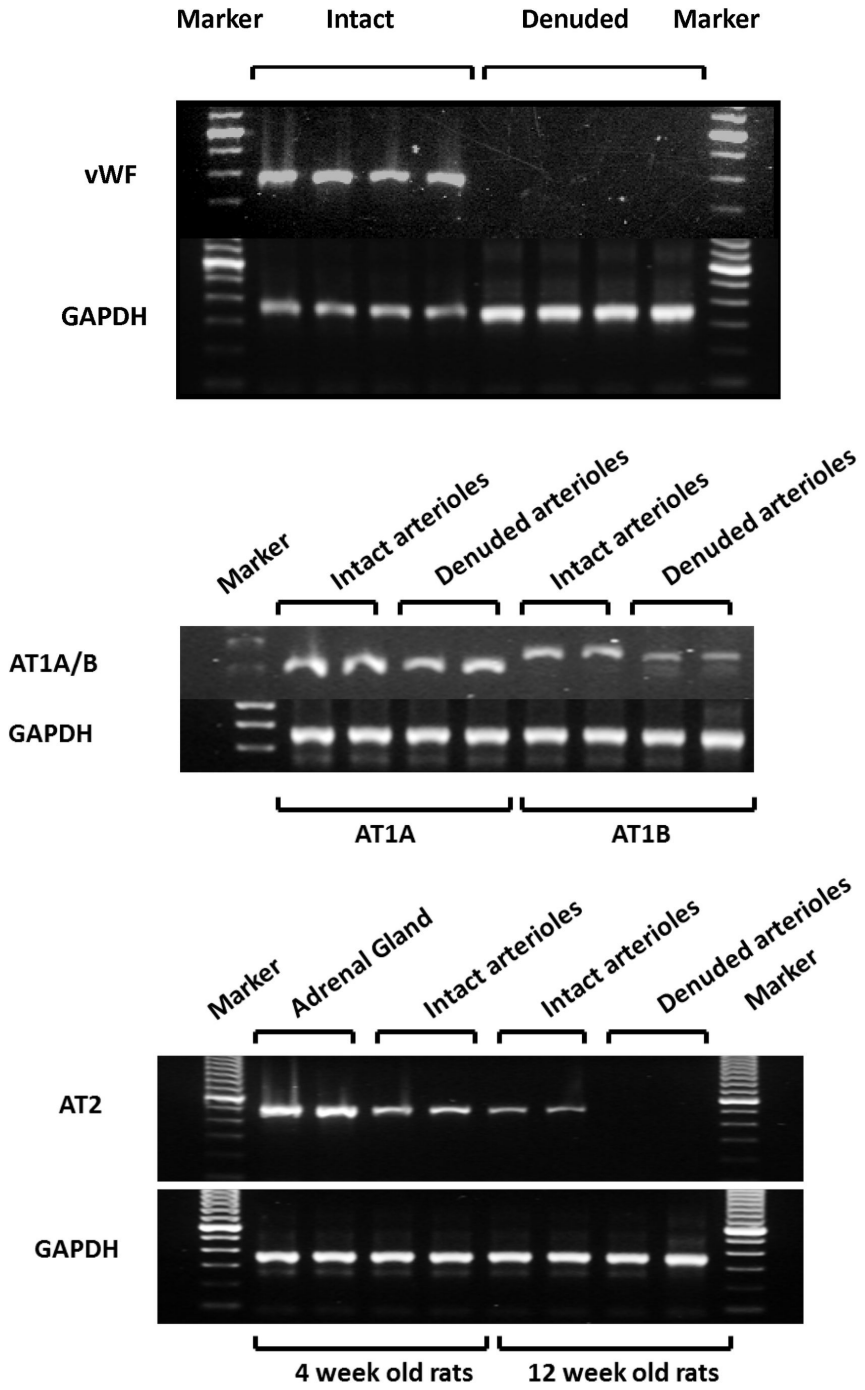
The expression of ANG II receptor subtypes in renal microvessels. The upper panel indicates the endothelium specific marker-von Willebrand factor (vWf) is expressed in intact vessels but not in vessels in which the endothelium was removed. Message for AT_1A_ and AT_1B_ receptors was detected by RT-PCR in both intact vessels and vessels with the endothelium removed indicating that they are expressed in VSM cells. Expression of the AT_2_ receptor was only detected in renal microvessels with an intact endothelium (lower panel). GAPDH was amplified in all of the samples.

RT-PCR analysis was performed to determine the ANG II receptor subtypes expressed in intact and endothelium-denuded renal microvessels. The efficiency of the removal of the endothelium was confirmed by measuring the expression of message for the endothelium specific marker-von Willebrand factor (vWf) [21,22]. The results of these experiments are presented in the upper panel of Figure 1. VWf message was highly expressed in intact but not endothelium-denuded renal microvessels. The control marker GAPDH was amplified in all the samples. These results indicate that the endothelial cells were effectively removed after perfusion of the renal microvessels with an anti-factor VIII antibody and complement. The results presented in the middle panel demonstrate that AT_1A_ and AT_1B_ receptors are expressed in both intact and denuded renal microvessels. In contrast, the expression of the AT_2_ receptor was only detected in intact vessels. These results suggest that both AT_1A_ and AT_1B_ receptors are expressed in renal VSM cells, however, AT_2_ receptor mRNA is expressed in the endothelium. These data are consistent with previous reports that the AT_2_ receptor is largely expressed in the endothelium rather than VSM cells [23-25]. 

### Effect of ANG II on the production of 20-HETE in renal microvessels

**Figure 2:**
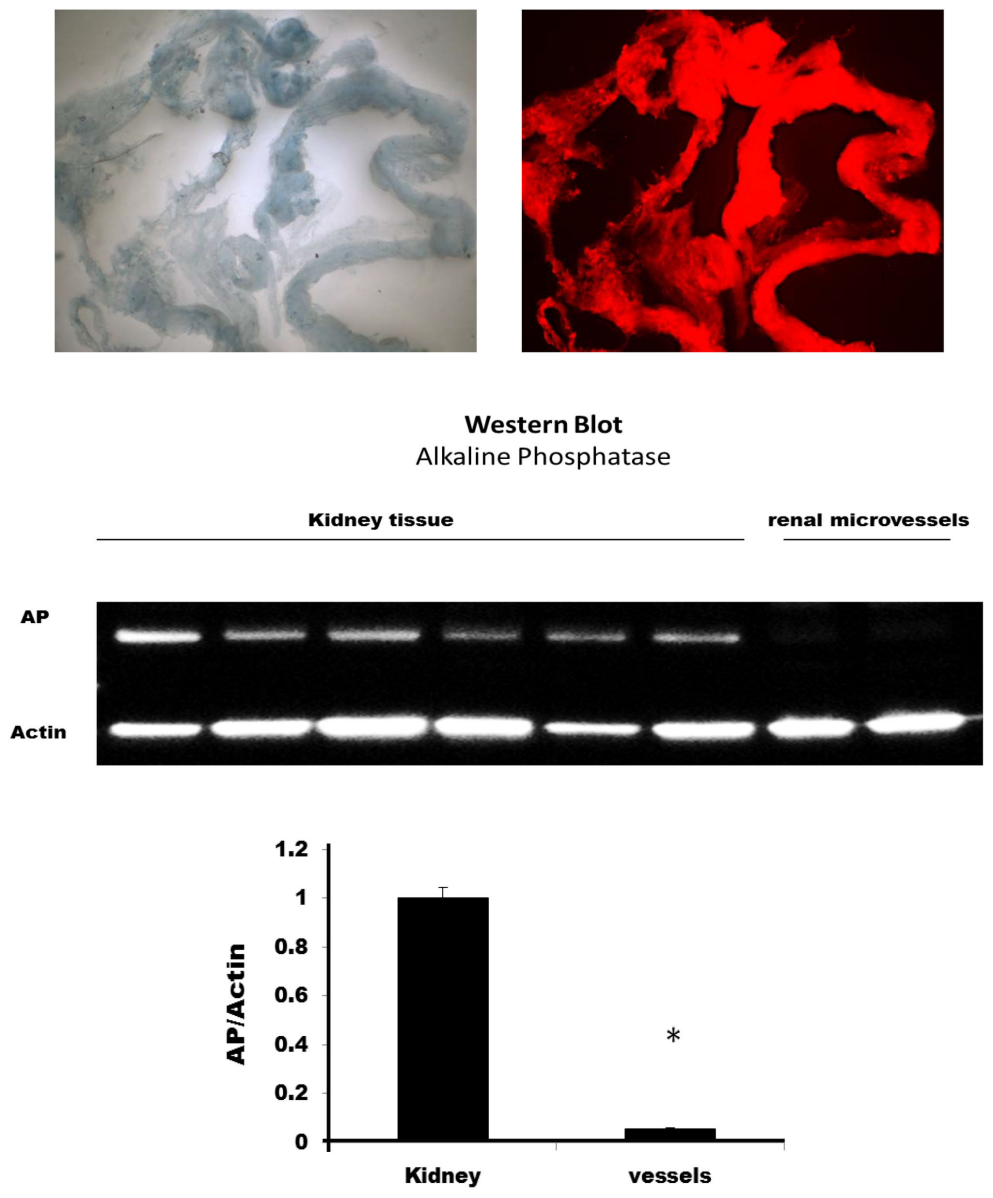
Typical appearance of the isolated renal microvessels. The upper left panel presents appearance of the vessels under white light illumination. The upper right panel presents the appearance of the vessels with excitation of 550 nm, emission of 610 nm. The vessels that were stained with Evans blue in the isolation procedure exhibit red fluoresce while the adherent tubules do not fluoresce. The lower panel compares alkaline phosphatase activity which is highly expressed in the proximal tubules in renal homogenates versus that seen in the renal microvessel preparation. * indicates a significant difference from control.

The typical appearance of the renal microvessels isolated by Evans blue sieving procedure is shown in the upper panel of Figure 2. The Evans blue stained vessels emit strong red fluorescence when excited using 550 nm light, whereas the unstained surrounding tubules are not fluorescent. The results indicate that all of the structures in the field exhibit strong red fluorescence indicating that there is little or no tubular contamination. The purity of the microvessel preparation was further confirmed by comparing the expression of alkaline phosphatase, which is highly expressed in renal proximal tubules, in renal homogenates versus that seen in the renal microvessel preparations [12]. The results presented in the lower panel of Figure 2 indicated that alkaline phosphatase activity was reduced by 95% in the renal microvessels relative to the expression seen in renal cortical homogenates. 

**Figure 3:**
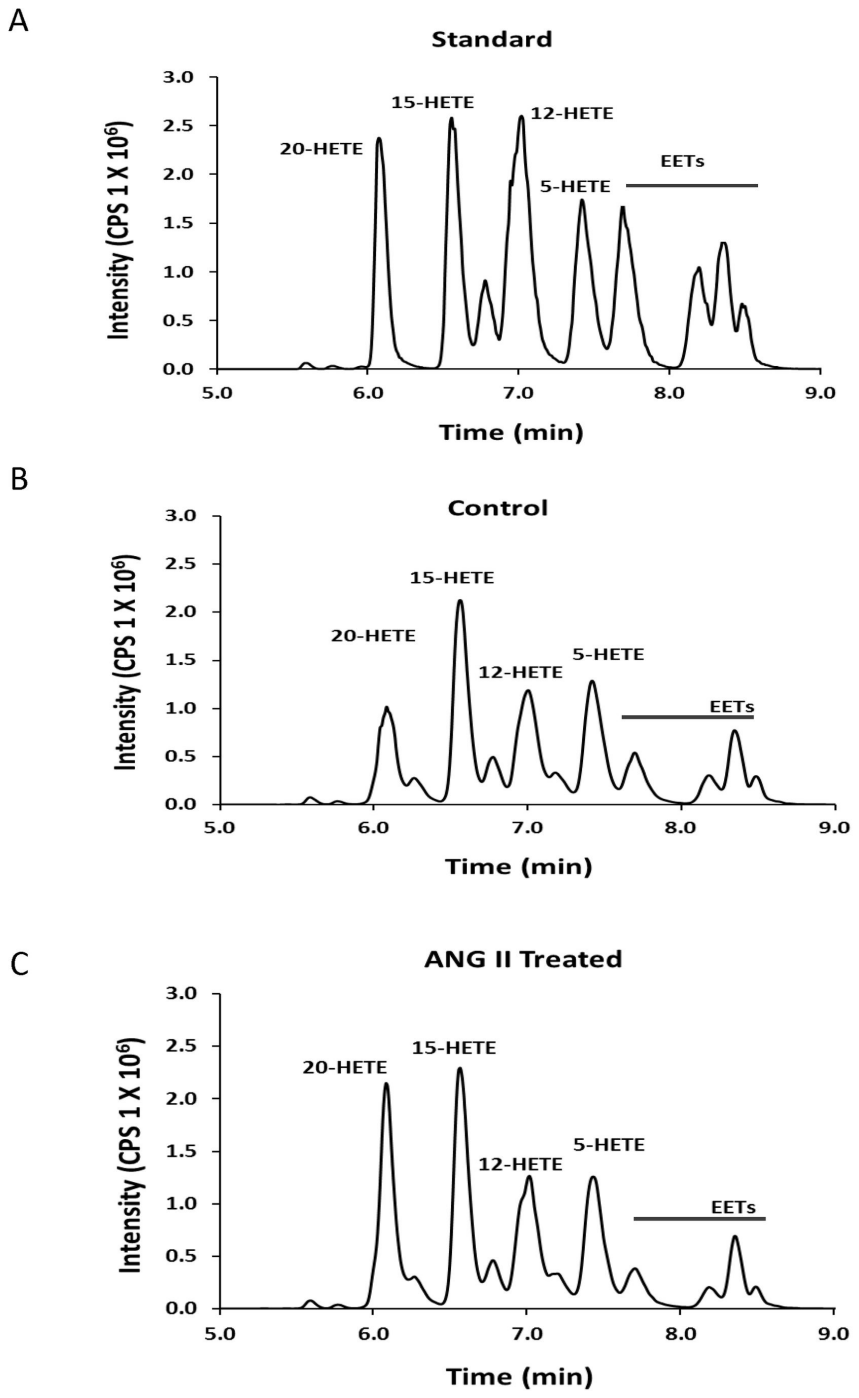
Production of 20-HETE in renal microvessels measured by LC/MS/MS. Panel A presents a typical chromatograph indicating the retention times of a mixture of 20 ng aliquots of various CYP450 metabolites of AA. Panel B presents a typical chromatograph of the metabolites of AA produced by control incubation of renal microvessels (0.68 mg protein). Panel C presents a typical chromatograph of the metabolites of AA produced when an aliquot of these same vessels were incubated with ANG II (0.66 mg protein).

The production of 20-HETE in renal microvessels was measured by liquid chromatography/mass spectrometry (LC/MS). Preliminary experiments were first performed to determine the effects of ANG II on the production and release of 20-HETE in renal microvessels in the presence and absence of exogenous AA. The results of these experiments indicate that ANG II increases the production of 20-HETE under both conditions, however, the response to ANG II was much greater in the presence of exogenous AA. A representative chromatogram showing the separation of 20 ng aliquots of various CYP eicosanoid standards is presented in Figure 3A. A representative chromatogram showing the metabolites of AA produced by renal microvessels before and after addition of ANG II are presented in Figure 3B and 3C. These results indicate that renal microvessels produce 5-, 12-, 15-, and 20-HETE along with various EETs under control conditions. ANG II increased the formation of 20-HETE in the vessels but it had little effect on the formation of other metabolites of AA. 

**Figure 4:**
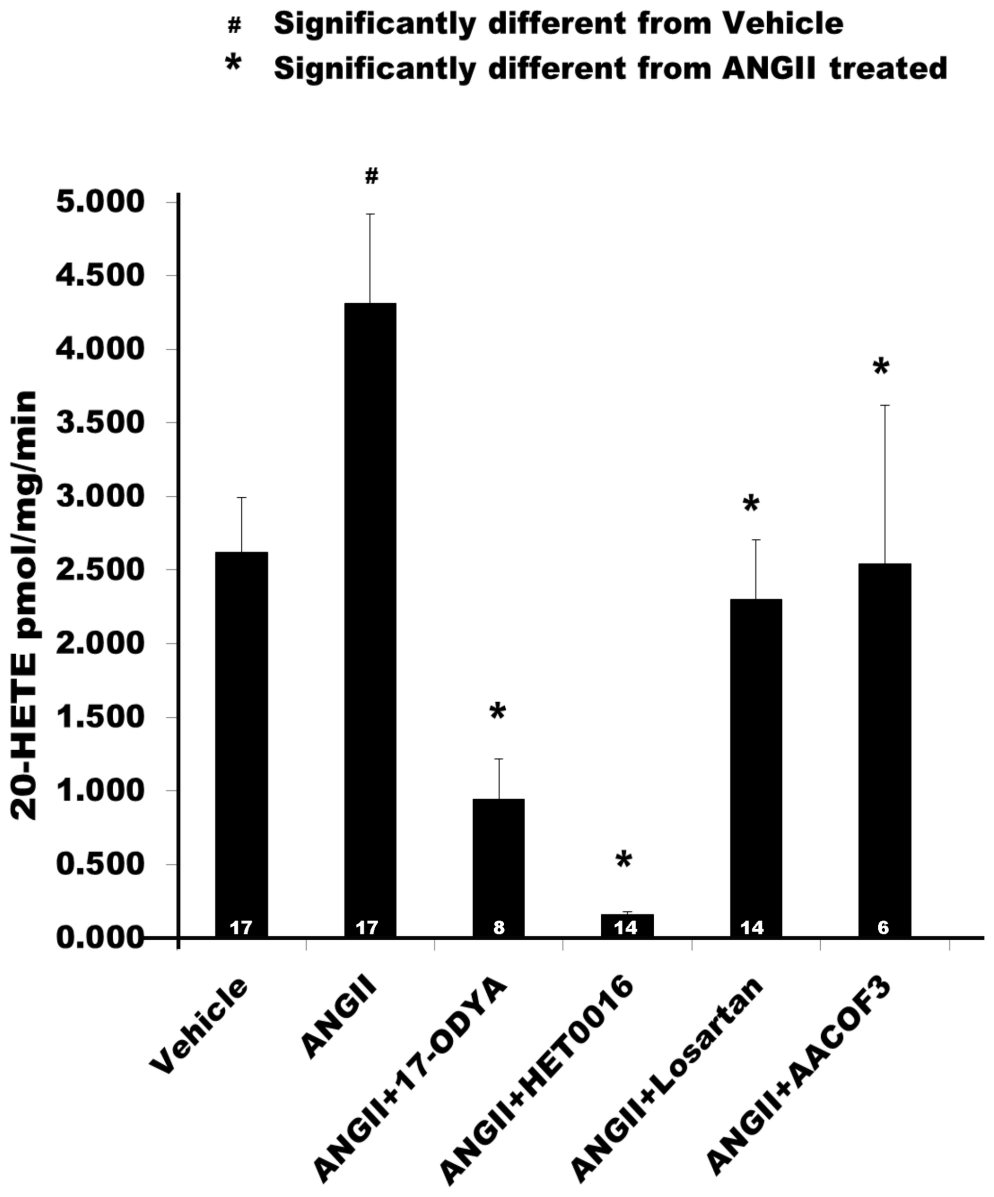
Effect of ANG II on the production of 20-HETE in renal microvessels. Comparison of the production of 20-HETE in renal microvessels treated with vehicle, ANG II and ANG II plus inhibitors of the synthesis of 20-HETE, 17-ODYA (10^-5^ M) and HET0016 (10^-6^ M), a phospholipase A_2_ inhibitor, AACOF_3_ (2 X 10^-5^ M), a phospholipase C inhibitor, of U-73122 (10^-5^ M), and the AT_1_ receptor blocker, Losartan (10^-6^ M). Numbers in the bars indicate the number of vessel preparations studied. # indicates a significant difference from the corresponding value in vessels treated with vehicle. * indicates a significant difference from the corresponding value in vessels treated with ANG II (10^-7^ M).

A summary of the effects of ANG II (10^-7^ M) on the production of 20-HETE in renal microvessels is presented in Figure 4. Treatment of renal microvessels with ANG II significantly increased the production of 20-HETE from 2.6 ± 0.4 to 4.3 ± 0.6 pmol/mg/min (n = 17, P < 0.05). It had no significant effect on the formation of EETs and other HETEs (Table 1). 17-ODYA is an inhibitor of the formation of both EETs and 20-HETE [1,26,27], while HET0016 is a more specific inhibitor of the formation of 20-HETE [28]. The stimulatory effect of ANG II on the production of 20-HETE was completely blocked by 17-ODYA (10^-5^ M), HET0016 (10^-8^ M), the PLA_2_ inhibitor, AACOF3 (2X10^-5^ M) and the AT_1_ receptor antagonist, Losartan (10^-6^ M). In contrast, blockade of the AT_2_ receptor with PD123319 (10^-7^ M, n=8) had no effect on ANG II-stimulated 20-HETE production (data not shown). These results indicate that treatment of renal microvessels with ANG II selectively increases the formation of 20-HETE in renal microvessels and the stimulatory effect of ANG II is mediated by activation of the AT_1_ receptor and a PLA_2_- dependent pathway.

**Table 1:** Summary of the production of various metabolites of arachidonic acid by renal microvessels under control conditions and after addition of ANG II (10^-7^ M).

	**Vehicle** pmol/mg/min	**ANG II** pmol/mg/min
**5-HETE**	1.43 ± 0.50	1.85 ± 0.33
**12-HETE**	2.30 ± 0.54	2.48 ± 0.60
**15-HETE**	1.48 ± 0.42	2.22 ± 0.38
**14,15-DiHETE**	1.07 ± 0.24	1.27 ± 0.19
**11,12-DiHETE**	2.26 ± 0.48	3.06 ± 0.47
**8,9-DiHETE**	1.20 ± 0.22	1.56 ± 0.25
**5,6-DiHETE**	0.02 ± 0.01	0.25 ± 0.13
**14,15-EET**	0.25 ± 0.10	0.47 ± 0.09
**11,12-EET**	0.23 ± 0.09	0.59 ± 0.19
**8,9-EET**	0.42 ± 0.22	0.71 ± 0.17
**5,6-EET**	0.30 ± 0.12	0.42 ± 0.09

The production of various metabolites of arachidonic acid by renal microvessels (N = 12-22 preparations) are presented. There was no significant difference in the production of any of the metabolites in the vehicle and ANG II treated vessels.

### Role of 20-HETE in the vasoconstrictor response to ANG II in rat renal microvessels

The contribution of 20-HETE to the vasoconstrictor response to ANG II was determined by comparing the response of renal interlobular arterioles to ANG II under control conditions and after blocking the formation of 20-HETE with 17-ODYA (10^-5^ M). In order to eliminate the influence of vasodilator mediators from the endothelium, the vessels were denuded by perfusion with anti-factor VIII antibody and complement as previously described [16] and the efficiency of the removal of the endothelium was confirmed by measuring the expression of message for the endothelium specific marker-von-Willebrand factor (vWf) [21,22]. The effectiveness of removal of the endothelium was also confirmed by measuring the response of the vessels to acetylcholine (Ach, 10^-6^ M). The control diameter of the vessels were 130 ± 7 μm. Addition of phenylephrine (1 μM) reduced the inner diameter by 50 % to 66 ± 5 μm. and Ach (10^-6^ M) had no significant effect on the diameter of these denuded renal arterioles (67 ± 5 μm, n=6). 

**Figure 5:**
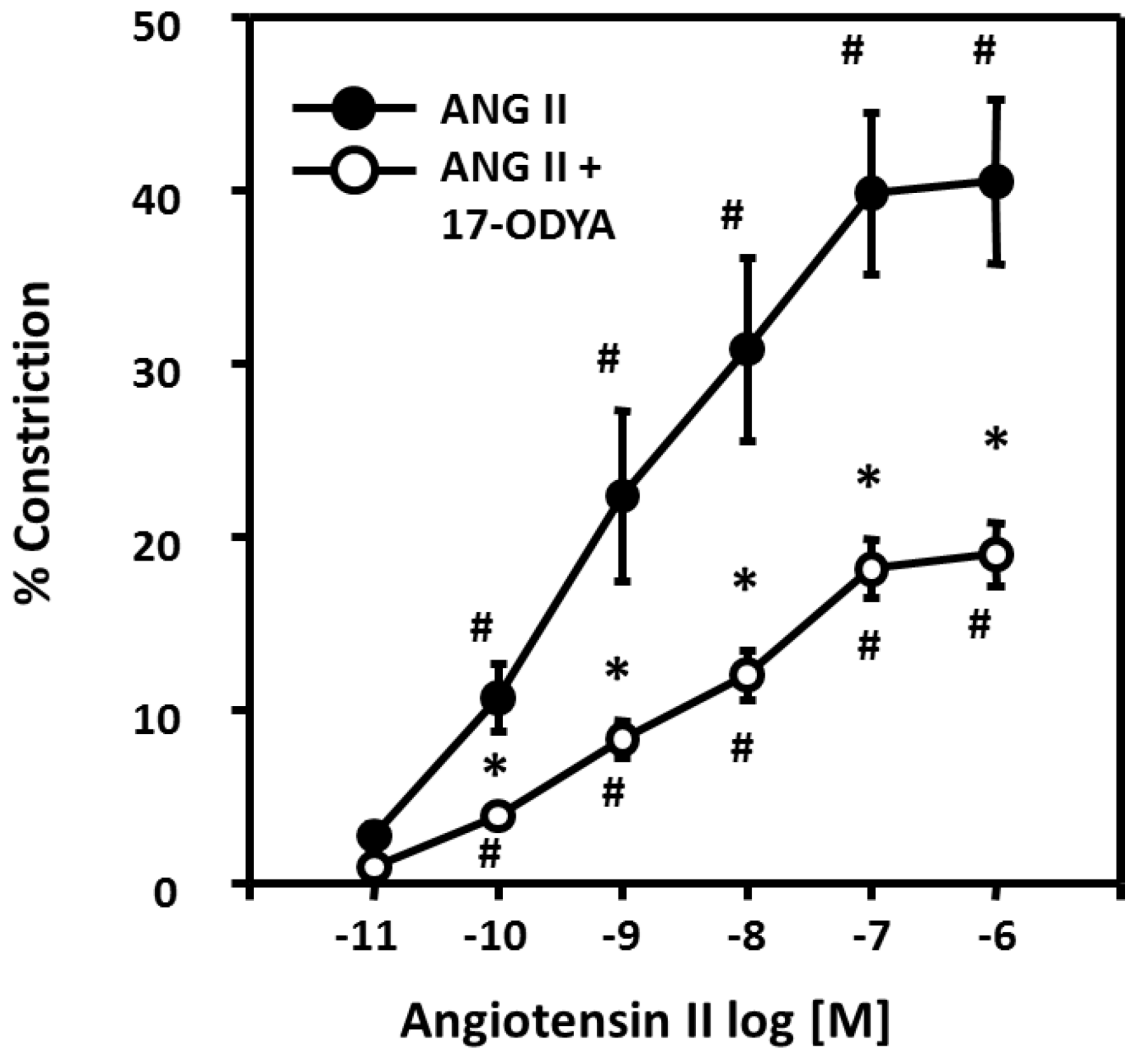
Effects of 20-HETE on vasoconstrictor responses to ANG II in renal microvessels. Concentration response curves for the vasoconstrictor response to ANG II (10^-11^ to 10^-6^ M) in pressurized renal microvessels with the endothelium removed (N=5) are presented before and after addition of 17-ODYA (10^-5^ M). 17-ODYA an inhibitor of the formation of 20-HETE had no significant effect on the EC50 but it reduced the maximal response to ANG II by 50%. # indicates a significant difference from the value at the lowest concentration of ANG II concentration (10^-11^ M). * indicates a significant difference from the corresponding value prior to treatment of 17-ODYA (10^-5^ M).

The effects of 17-ODYA on the vasoconstrictor response to ANG II in the denuded renal microvessels are summarized in Figure 5. ANG II decreased the inner diameter of the denuded renal interlobular arterioles in a concentration dependent manner by 41 ± 5 %. After addition of the 20-HETE inhibitor 17-ODYA (10^-5^ M), the EC50 for ANG II was not significantly altered but the maximal vasoconstrictor response was reduced by 50%.

### Effect of ANG II on K_Ca_ channel activity in renal VSM cells

**Figure 6:**
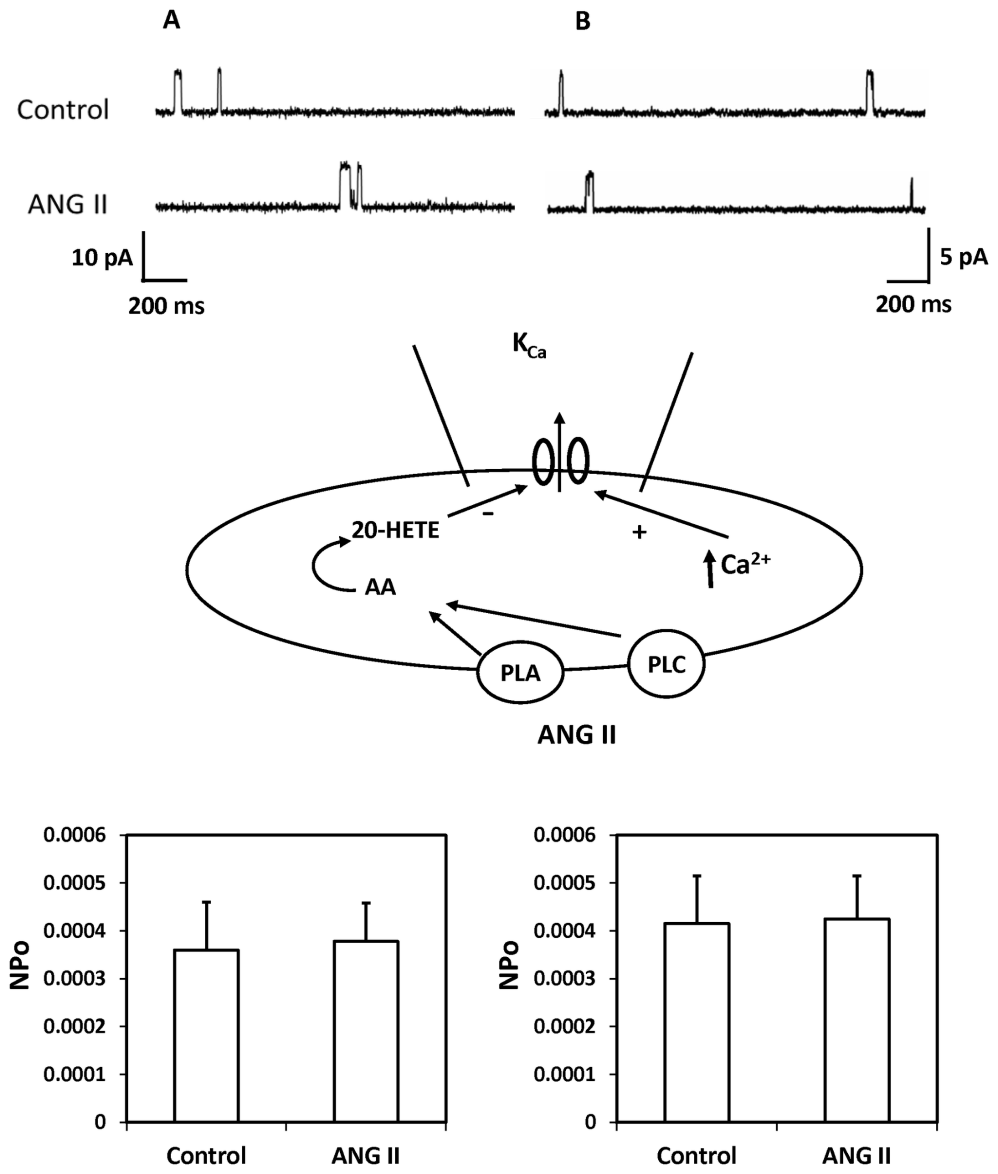
Effect of ANG II on the K_Ca_ channel activity in renal VSM cells. The upper panel presents a representative current recording of the effects of ANG II on of the K_Ca_ channel activity recorded using a cell-attached patches of VSM cells freshly isolated from renal microvessels at a pipette potential -40 mV in high K^+^ solution with 140 mM KCl in the pipette to null membrane potential (panel A) or in normal PSS with 140 mM KCL in the pipette at the resting membrane potential (pipette potential = 0 mV, panel B). The middle panel summarizes the cell-attached patch clamp recording mode. The lower panel presents a summary of the effects of ANG II on the activity of K_Ca_ channel recorded in depolarized cells (panel A) in a high K+ solution and in cells bathed in PSS at a normal depolarized membrane potential (panel B). Recordings were obtained from 6-8 cells under each condition.

A representative recording depicting the effects of ANG II on single-channel K^+^ currents recorded using cell-attached patches on freshly isolated renal arteriolar VSM cells is presented in Figure 6. The single K_Ca_ channel currents were recorded with the cells bathed in a high K^+^ solution to null membrane potential at pipette potential of –40 mV in panel A or in normal PSS with 140 mM KCl in the pipette at the resting membrane potential (pipette potential = 0 mV) in panel B. ANG II (10^-7^ M) did not alter the NPo of K_Ca_ channels under either of these conditions. ANG II also had no effect on K_Ca_ single channel amplitude which averaged 9.6 ± 0.1 before and 9.5 ± 0.2 pA after addition of ANG II to the bath. 

### Effect of ANG II on [Ca^2+^]_i_ of renal VSM cells

**Figure 7:**
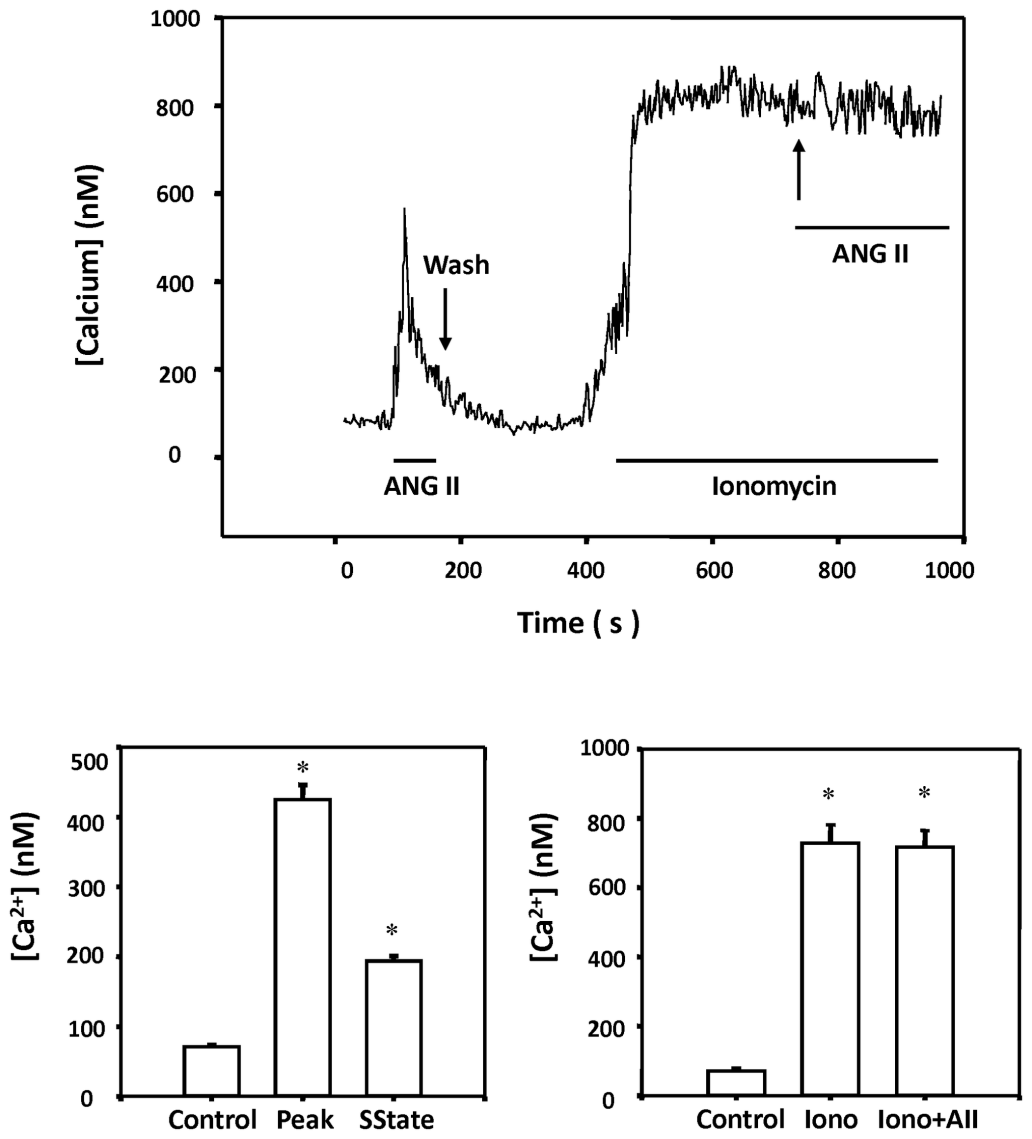
Effect of ANG II on intracellular calcium response in renal VSM cells. The upper panel presents a representative tracing of a [Ca^2+^]_i_ response of a single renal microvessel VSM cell to ANG II (10^-7^ M) bathed in PSS before and after addition of ionomycin (10^-6^ M). ANG II elicited a rise in [Ca^2+^]_i_ in renal VSM cells under control conditions. Addition of ionomycin raised baseline [Ca^2+^]_i_ and under these conditions ANG II had no additional effect on [ Ca^2+^]_i_. The lower left panel summarizes the effects of on peak and plateau [Ca^2+^]_i_ responses to ANG II (10^-7^ M) in the renal VSM cells. The right panel presents the [Ca^2+^]_i_ response to ANG II after addition of ionomycin. * indicates a significant rise in [Ca^2+^]_i_ over baseline. Mean values + SE recorded from 45-70 cells (5-10 cells per experiment) isolated from 9 different renal VSM cell isolation are presented.

The effects of ANG II on [Ca^2+^]_i_ was examined in renal VSM cells bathed in normal PSS in the presence and absence of the calcium ionophore, ionomycin (10^-6^ M). The results of these experiments are presented in Figure 7. Baseline [Ca^2+^]_i_ of VSM cells averaged 71 ± 3 nM. ANG II (10^-7^ M) increased [Ca^2+^]_i_ to a peak value of 425 ± 21 nM that returned to an elevated level of 194 ± 7 nM. 100-200 msec after administration of ANG II. In another group of cells, addition of ionomycin produced a large sustained increase of [Ca^2+^]_i_ from 90 ± 9 nM to 728 ± 53 nM. In the presence of ionomycin, ANG II had no effect on [Ca^2+^]_i_. 

### Effect of ANG II on K_Ca_ channel activity response in renal VSM cells in the presence of ionomycin

**Figure 8:**
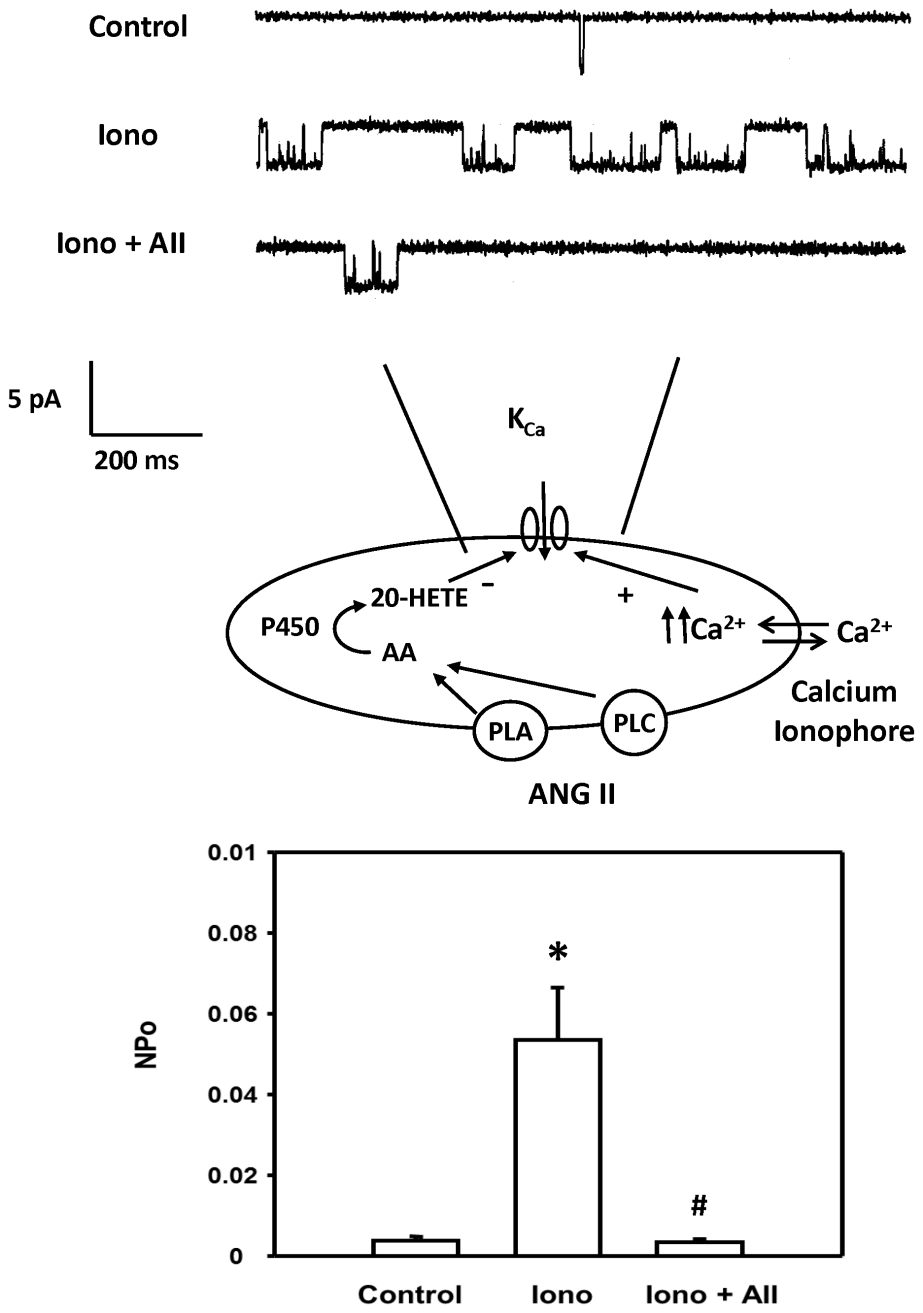
Effect of ANG II on K_Ca_ channel activity in renal VSM cells with high [Ca^2+^]_i_. The upper panel presents a representative current recording of the effects of ANG II on K_Ca_ channel activity recorded using cell-attached patches on renal arteriolar VSM cells. The middle panel summarizes the patch clamp recording mode. K_Ca_ channel currents were recorded with 140 mM KCl in the pipette at the resting membrane potential (pipette potential = 0 mV) and the cells were bathed in normal physiological salt solution in the presence of ionomycin. The lower panel summarizes the effects of ionomycin and subsequent addition of ANG II on the K_Ca_ channel activity. * indicates the significant difference from the control value. # indicates a significant difference from the corresponding value recorded in the presence of ionomycin.

The effects of ANG II on K_Ca_ channel activity in renal arteriolar VSM cells bathed in normal PSS recorded in the cell attached mode at resting membrane potential (0 mv pipette potential) is presented in Figure 8. The baseline activity of this channel was very low (NPo, 0.0004 ± 0.0001). Raising [Ca^2+^]_i_ with ionomycin (10^-6^ M) increased channel activity 10-fold. But, with the addition of ANG II (10^-7^ M) to the bath in the presence of ionomycin reduced K_Ca_ channel activity by 93 ± 3%. 

**Figure 9:**
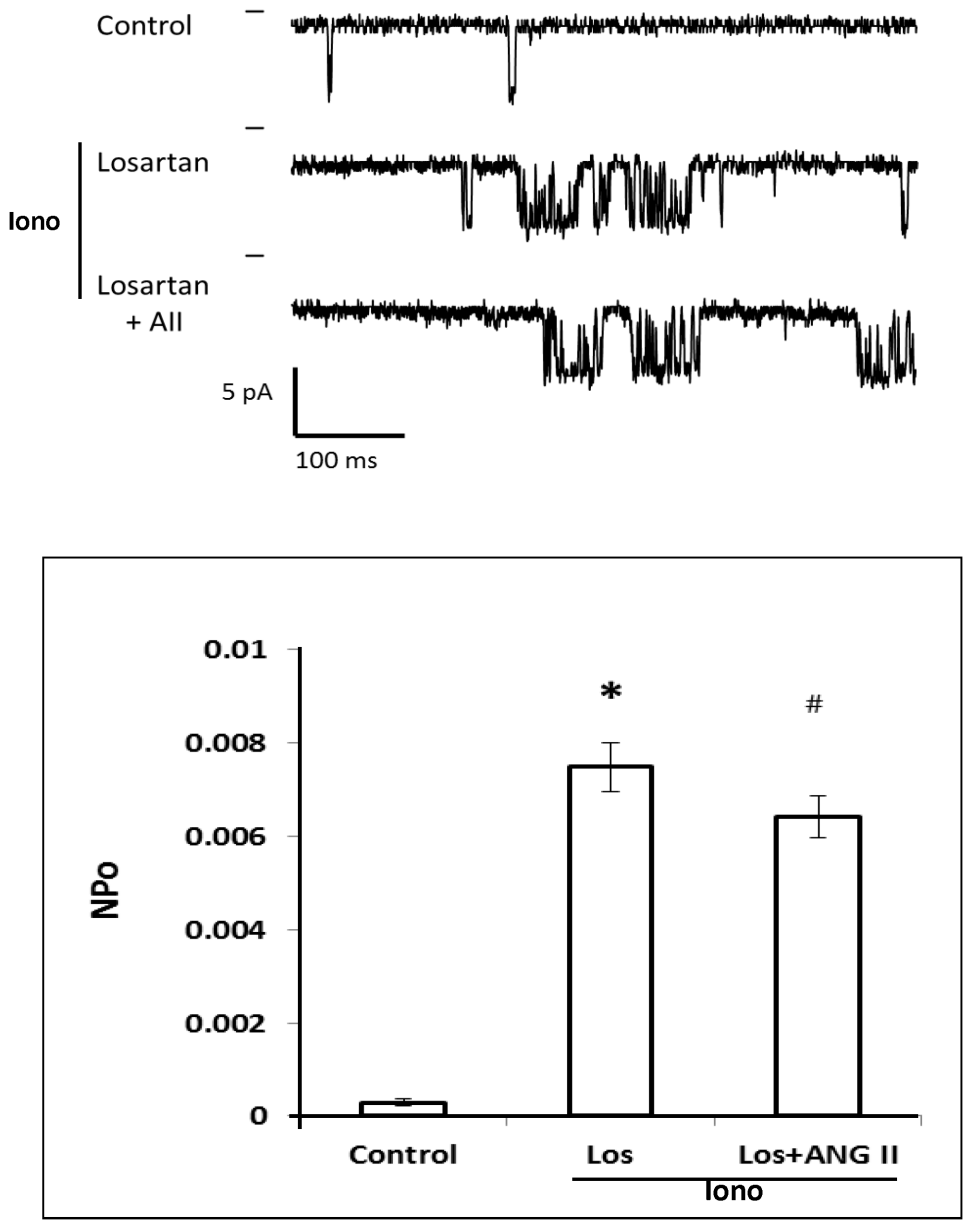
Effect of Losartan on the inhibitory action of ANG II on K_Ca_ channel activity in renal VSM cells. The upper panel presents a representative current traces recording of the effects of Losartan and ANG II on the K_Ca_ channel activity recorded in the presence of ionomycin. K_Ca_ channel currents were recorded with 140 mM KCl in the pipette at the resting membrane potential (pipette potential = 0 mV) and the cells were bathed in normal physiological salt solution in the presence of ionomycin. The lower panel summarizes the effects of Losartan and ANG II on K_Ca_ channel activity recorded in the cell attached mode in the presence of ionomycin in the bath. * indicates a significant difference from the control value recorded from the same cells. # indicates a significant difference from the corresponding value recorded in the presence of ionomycin.

We next determined which ANG II receptor contributes to the inhibitory action of ANG II on K_Ca_ channel activity. The results of these experiments are presented in Figure 9. The addition of ionomycin produced a 10-fold increase in K_Ca_ channel activity. In the presence of the AT_1_ receptor antagonist, Losartan, ANG II had no effect on K_Ca_ channel activity. 

**Figure 10:**
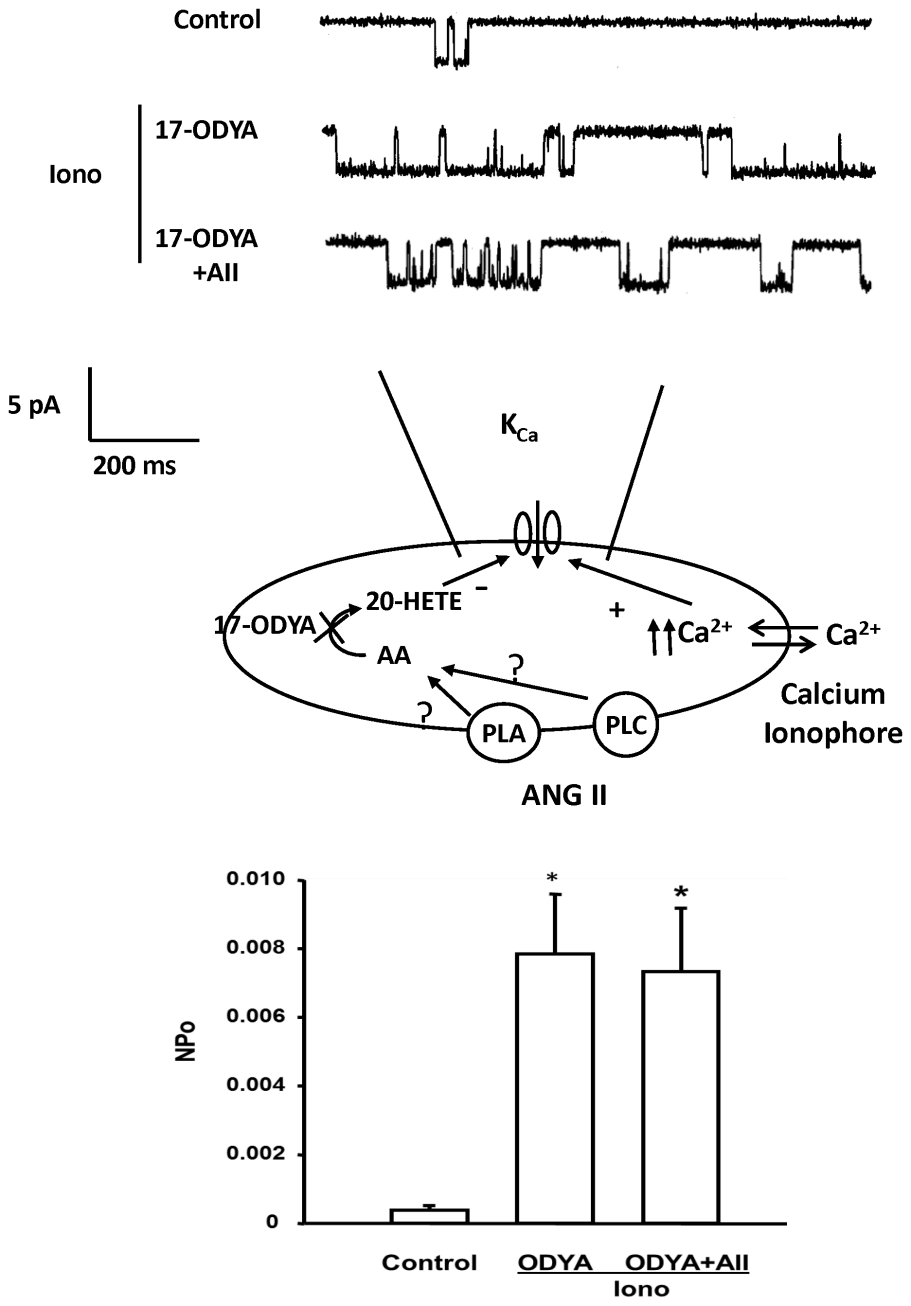
Effect of 17-ODYA on the inhibitory action of ANG II on K_Ca_ channel activity in renal VSM cells. Upper panel presents a representative tracing K_Ca_ channel activity in the cell-attached mode before and after addition of 17-ODYA (10^-7^ M) and 17-ODYA plus ANG II to the bath. K_Ca_ channel currents were recorded with 140 mM KCl in the pipette at the resting membrane potential (pipette potential = 0 mV) and the cells were bathed in normal physiological salt solution in the presence of ionomycin. The middle panel summarizes the patch clamp recording mode. Lower panel summarizes the effects of 17-ODYA and 17-ODYA and ANG II on the open probability of the K_Ca_ channel in renal VSM cells (n = 4 cells from 4 rats). * indicates a significant difference versus the corresponding control value.

The effect of ANG II on K_Ca_ channel activity was also studied before and after blockade of 20-HETE formation with 17-ODYA. The representative traces are shown in the upper panel of Figure 10. Addition of 17-ODYA (10^-5^ M) and ionomycin (10^-6^ M) to the bath solution significantly increased the NPo of the K_Ca_ channel by 20-fold. Subsequent addition of ANG II had no significant effect on K_Ca_ channel activity after the synthesis of 20-HETE in renal VSM was blocked by 17-ODYA.

### Role of PLC in ANG II induced-K_Ca_ channel inhibition

**Figure 11:**
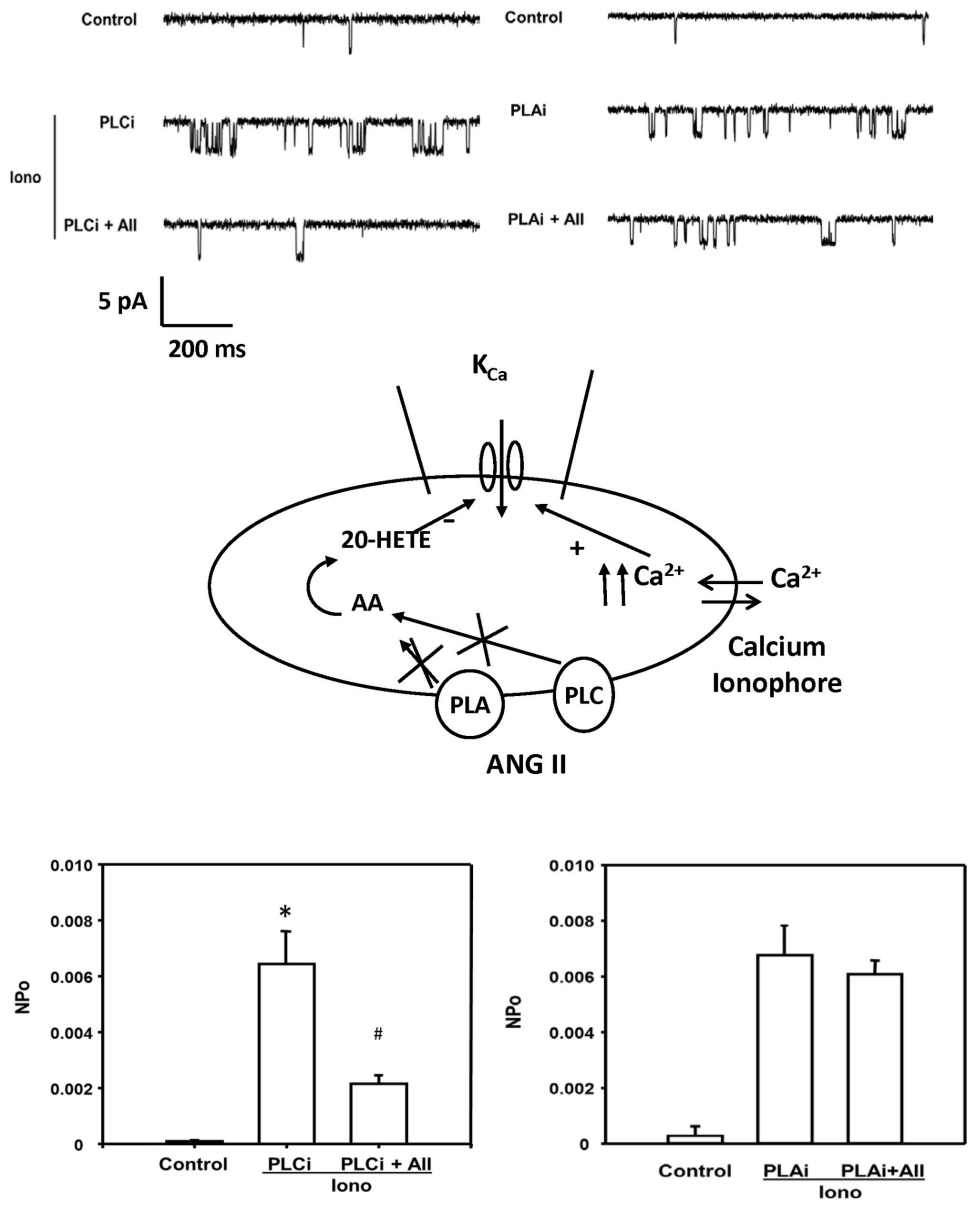
Effect of blockade of PLC and PLA_2_ on the inhibitory action of ANG II on K_Ca_ channel activity in renal VSM cells. The upper panel presents representative tracings of single K_Ca_ channel currents recorded at the resting membrane potential (pipette potential = 0 mV) in the cell-attached mode before and after addition of PLC inhibitor U-73122 (10^-5^ M) and U-73122 plus ANG II (10^-7^ M) to the bath (left). The effects of the PLA_2_ inhibitor, AACOF_3_ (2 x 10^-5^ M) and AACOF_3_ plus ANGII (10^-7^ M) are presented in the right panel. K_Ca_ channel currents were recorded with 140 mM KCl in the pipette at the resting membrane potential (pipette potential = 0 mV) and the cells were bathed in normal physiological salt solution in the presence of ionomycin. The middle panel summarizes the patch clamp recording mode. K_Ca_ channel currents were recorded as described in Figure 8. The lower left panel summarizes the effect of U-73122 and ANG II on the open probability of the K_Ca_ channel in renal VSM cells while the lower right panel depicts the effects of AACOF_3_ on the response to ANGII. * indicates a significant difference versus control and # indicates a significant difference from the corresponding value recorded after PLC activity was inhibited with U-73122 or PLA2 activity was inhibited with AACOF3.

Previous studies have indicated that ANG II stimulates PLC activity following activation of the AT_1_ receptor coupled to a Gq signal transduction pathway [29]. To test the role of PLC in mediating the inhibitory action of ANG II on K_Ca_ channel activity, single K_Ca_ channel currents were recorded from VSM cells before and after addition of ionomycin (10^-6^ M) and the selective PLC inhibitor, U-73122 (10^-5^ M) to the bath and then after addition of ANG II (10^-7^ M). Addition of ionomycin and U-73122 significantly increased the activity of K_Ca_ channel by 6-fold. In the presence of the ionomycin and U-73122, ANG II reduced the NPo of the K_Ca_ channel by 62% (Figure 11, left panel). 

### Effects of PLA_2_ on the inhibitory effect of ANG II on K_Ca_ channel activity

We also examined the role of PLA_2_ on ANG II-induced inhibitory effect on K_Ca_ channel currents in VSM cells. The K_Ca_ channel currents were recorded before and after addition of ionomycin (10^-6^ M) and the selective PLA_2_ inhibitor, AACOF_3_ (2 X 10^-5^ M) to the bath followed by ANG II (10^-7^ M). The results are presented in the right panel of Figure 11. Addition of AACOF_3_ and ionomycin to the bath increased K_Ca_ channel activity 4-fold. Under these conditions, addition of ANG II did not significantly change the NPo of the K_Ca_ channel.

### Effect of 17-ODYA on the effects of ANG II on [Ca^2+^]_i_

**Figure 12:**
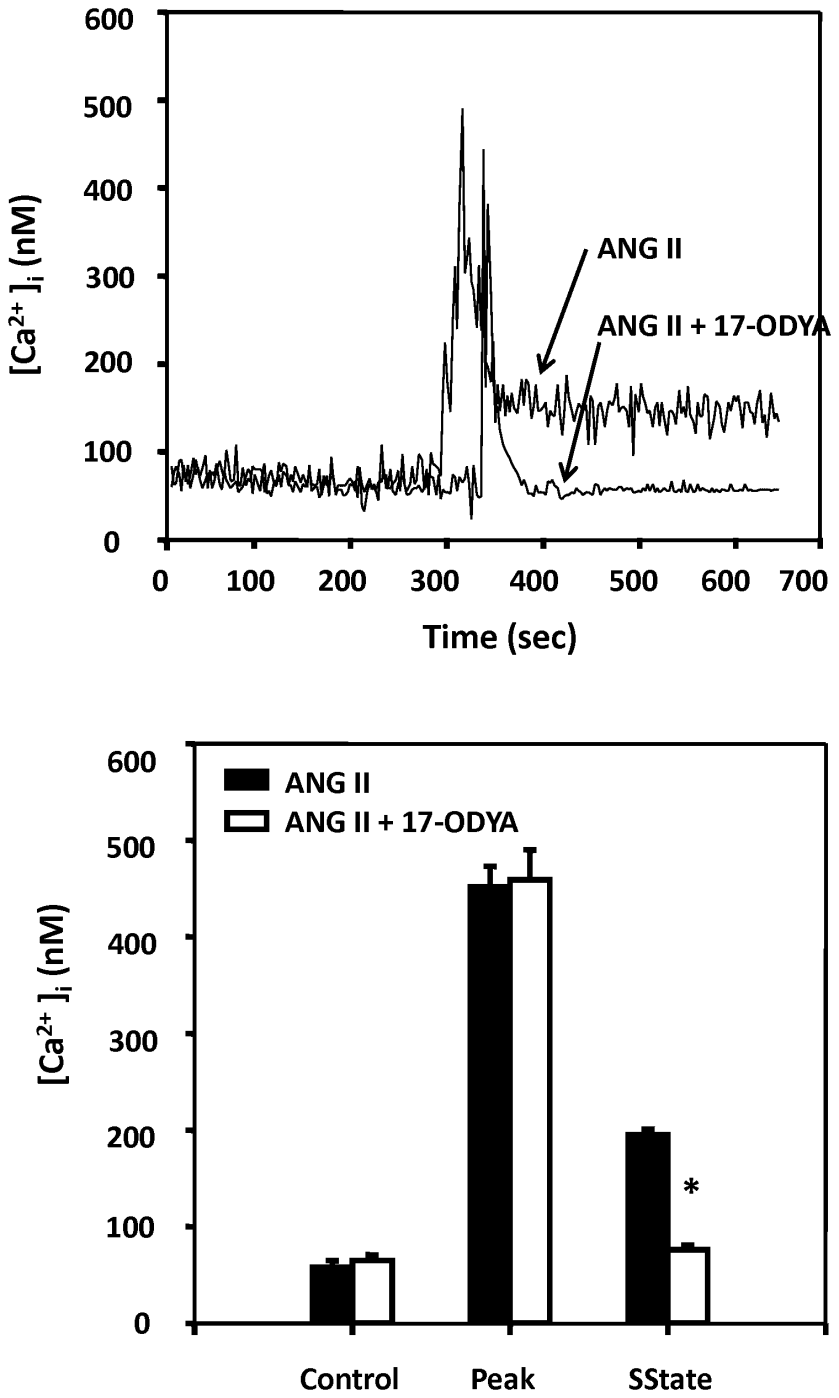
Effects of ANG II on [Ca^2+^]_i_ in renal VSM cells before and after 17-ODYA. The upper panel presents representative tracings of the effect of ANG II on [Ca^2+^]_i_ in renal VSM before and after blockade of the synthesis of 20-HETE with 17-ODYA (10^-5^ M). The lower panel presents the peak and steady state [Ca^2+^]_i_ responses to ANG II measured before and after addition of 17-ODYA to the bath. Mean values + SE recorded from 25-30 cells (5-8 cells per experiment) isolated from 6 different renal VSM isolations are presented. * indicates a significant difference between ANG II and ANG II plus 17-ODYA treated vessels.

The effect of blockade of the synthesis of 20-HETE with 17-ODYA on the effects of ANG II on [Ca^2+^]_i_ in freshly isolated renal arteriolar VSM cells is presented in Figure 12. ANG II (10^-7^ M) induced a rapid increase in [Ca^2+^]_i_ from 58 ± 7 to 452 ± 21 nM that was followed by a recovery to a steady-state level of 195 ± 6 nM. After administration of 17-ODYA (10^-5^ M), the peak response to ANG II was similar to that seen in control cells (from 65 ± 6 to 459 ± 31 nM), however, the steady-state response to ANG II was markedly reduced to (76 ± 5 nM). 

## Discussion

Previous studies have indicated that ANG II increases the release of AA in VSM cells by activation of PLA_2_ and/or PLC [4-6]. AA is a substrate for the formation of 20-HETE in renal arterioles and 20-HETE has been reported to constrict renal and cerebral arteries through depolarization of VSM cells by blocking the large conductance K_Ca_ channel [7,30,31]. However, the role of 20-HETE in modulating its vasoconstrictor actions of ANG II by affecting K_Ca_ channel activity remains to be determined. Therefore, the present study examined the role of 20-HETE in mediating the inhibitory effects of ANG II on K_Ca_ channel activity in rat renal VSM cells.

The effects of ANG II on the production of 20-HETE in renal microvessels were first studied utilizing LC/MS. The results indicate that ANG II selectively increases 20-HETE production in renal microvessels but it has little effect on the production of EETs and other metabolites of AA. This effect was abolished by the AT_1_ receptor blocker Losartan, whereas administration of the AT_2_ receptor antagonist had no effect on the ability of ANG II to stimulate the production of 20-HETE. In the presence of calcium ionophore to fix intracellular calcium concentration at a high level, ANG II reduced K_Ca_ channel activity in freshly isolated rat renal VSM cells. The inhibitory effect of ANG II was blocked by administration of 17-ODYA which is a specific inhibitor of the production of 20-HETE in renal VSM cells. These results are consistent with previous reports that 20-HETE has a direct effect to inhibit the K_Ca_ channel activity in renal VSM cells even though it increases intracellular calcium levels which normally would activate these channels [7,32]. 

The present results indicating that ANG II stimulates production of 20-HETE in renal microvessels is also consistent with previous findings in our lab [17,31] and others [4,5]. In this regard, Croft et al (2000) reported that the effect of ANG II on the endogenous production of 20-HETE in renal microvessels was mediated by the AT_2_ receptor and could be blocked by PD-123319 at a concentration of 10^-4^ M, but not by AT_1_ receptor antagonist Losartan (10^-4^ M). In contrast, the results of the present study indicate that a much lower dose of losartan (10^-6^ M) was effective in blocking the effects of ANG II on the production of 20-HETE, whereas PD-123319 (10^-7^ M) had no effect. The difference in the results may be due to the different concentrations of the AT_1_ and AT_2_ receptor blockers used in the two studies. High concentration of PD-123319 (10^-4^ M) have been reported to block both AT_1_ and AT_2_ receptors [33]. Another difference is that we studied the ability of ANG II to stimulate the production of 20-HETE following addition of a saturating concentration of the substrate AA to the bath to eliminate the potential influence of release of preformed 20-HETE, whereas the studies by Croft et al [4] were done in the absence of substrate and focused on the formation and release of 20-HETE from endogenous phospholipid pools. 

 In the present study pretreatment of the renal microvessels with 17-ODYA, an inhibitor of the formation of both EETs and 20-HETE [1,26,27] prevented the increase in 20-HETE levels in isolated renal microvessels treated with ANG II. Administration of a more specific inhibitor of the synthesis of 20-HETE, HET0016 [28] had a similar effect and reduced the formation of 20-HETE by > 95%. These results indicate that ANG II increases the formation of 20-HETE in renal vascular smooth muscle by stimulating synthesis from AA rather than releasing preformed 20-HETE from phospholipid pools.

We also examined the effect of inhibitors of PLC and PLA_2_ on the ability of ANG II to increase the formation of 20-HETE in renal microvessels. Both inhibitors of PLC and PLA_2_ reduced 20-HETE levels in response to ANG II, however the PLA_2_ inhibitor had a greater effect than the PLC inhibitor. Since the vessels were incubated in the presence of a saturating concentration of the substrate AA, inhibition of the release of AA from membrane phospholipid pools does not account for these findings. Rather, we suggest that 20-HETE, once formed, is likely rapidly reincorporated into phospholipid pools in renal VSM and ANG II and sustained activation of PLC and PLA_2_ promotes the release of 20-HETE from these pools essentially opposing the reuptake process. According to this view, the PLA_2_ and PLC inhibitors likely reduced free 20-HETE levels in the vessels by promoting reuptake into membrane phospholipid pools. . 

The functional significance of the effects of 20-HETE on the inhibitory action of ANG II on the K_Ca_ channel to the vasoconstrictor response to ANG II was studied in pressurized renal interlobular arterioles. The endothelium was removed to reduce the modulation of the vasoconstrictor response by release of NO, prostaglandins and EETs secondary to stimulation of the AT_2_ receptor on the endothelium [34-36]. ANG II dose-dependently decreased the diameter of renal interlobular arteries by 40%, however, in the presence of the 17-ODYA, the maximum vasoconstrictor response to ANG II was reduced by 50%. These results indicating that 20-HETE potentiates the vasoconstrictor response to ANG II in renal interlobular arteries are consistent with previous findings of Alonso-Galicia et al (2002), who reported that administration of a 20-HETE inhibitor attenuated the acute pressor response to ANG II in rats in vivo by about 50% and chronic blockade attenuated the development of ANG II induced hypertension [1]. 

Patch clamp studies were performed to explore the mechanism by which 20-HETE potentiates the renal vasoconstrictor response to ANG II. The results of these experiments confirmed previous findings of Inscho et al and Fellner and Arendshorst that ANG II produces a large transient increase [Ca^2+^]_i_ in renal VSM cells [37,38]. However, ANG II had no significant effect on the activity of the K_Ca_ channel in renal arteriolar VSM recorded in the cell attached mode using a pipette potential of -40 mv with the cells bathed in a high K^+^ solution to null the membrane potential or in VSM cells bathed in PSS solution at a physiological resting membrane potential (pipette potential = 0 mV). This finding is surprising since ANG II increased [Ca^2+^]_i_ which should increase K_Ca_ channel activity in renal VSM [39] unless some other factor intervened to oppose this effect. This finding raised the possibility that ANG II may block the activation of K_Ca_ channel in VSM that normally would be expected to attenuate the vasoconstrictor response to ANG II by hyperpolarizing the cell and blocking subsequent calcium entry via voltage gated calcium channels.

To address this hypothesis, renal VSM cells were treated with the calcium ionomycin to clamp [Ca]_i_ at high levels to activate the K_Ca_ channels prior to administration of ANG II. Ionomycin raised [Ca^2+^]_i_ and markedly increased K_Ca_ channel activity. Subsequent administration of ANG II reduced the activity of the K_Ca_ channel by >90%. Blockade of the formation of 20-HETE with 17-ODYA, blockade of the AT_1_ receptor with Losartan or administration of inhibitors of PLC and PLA_2_ opposed the inhibitory effects of ANG II on the K_Ca_ channel. In other experiments, we found that pretreatment of renal VSM cells with 17-ODYA had no effect on the peak [Ca^2+^]_i_ response to ANG II but it reduced the sustained elevation in [Ca^2+^]_i_. This result is consistent with previous reports that the sustained [Ca^2+^]_i_ response to ANG II is dependent of membrane depolarization and calcium entry via voltage-sensitive calcium channels [7,32]. 

 In summary, the results of the present study indicates that ANG II constricts renal VSM cells by binding to AT_1_ receptors expressed on the VSM cells which activates of PLA_2_ to release AA from membrane phospholipid pools. AA is then converted into 20-HETE by CYP450 enzymes of the 4A family in VSM cells. 20-HETE then acts to inhibit the K_Ca_ channels, which in turn hyperpolarizes the VSM cells and opposes calcium entry through voltage gated calcium channels. The exact mechanism by which 20-HETE inhibits opening of K_Ca_ channels remains to be determined. However, previous studies have suggested that that it most likely involves activation of PKC [19,40,41], tyrosine kinase [19,42] and/or the Rho kinase [43] pathways.

## References

[1] Alonso-Galicia M, Maier KG, Greene AS, Cowley AW Jr., Roman RJ (2002) Role of 20-hydroxyeicosatetraenoic acid in the renal and vasoconstrictor actions of angiotensin II. Am J Physiol Regul Integr Comp Physiol 283: R60-R68. PubMed: 12069931. 12069931 10.1152/ajpregu.00664.2001

[2] Kohagura K, Arima S, Endo Y, Chiba Y, Ito O et al. (2001) Involvement of cytochrome P450 metabolites in the vascular action of angiotensin II on the afferent arterioles. Hypertens Res 24: 551-557. doi:10.1291/hypres.24.551. PubMed: 11675950. 11675950

[3] Imig JD, Zou AP, Ortiz de Montellano PR, Sui Z, Roman RJ (1994) Cytochrome P-450 inhibitors alter afferent arteriolar responses to elevations in pressure. Am J Physiol 266: H1879-H1885. PubMed: 8203587. 8203587 10.1152/ajpheart.1994.266.5.H1879

[4] Croft KD, McGiff JC, Sanchez-Mendoza A, Carroll MA (2000) Angiotensin II releases 20-HETE from rat renal microvessels. Am J Physiol Renal Physiol 279: F544-F551. PubMed: 10966934. 10966934 10.1152/ajprenal.2000.279.3.F544

[5] Chu ZM, Croft KD, Kingsbury DA, Falck JR, Reddy KM et al. (2000) Cytochrome P450 metabolites of arachidonic acid may be important mediators in angiotensin II-induced vasoconstriction in the rat mesentery in vivo. Clin Sci (Lond) 98: 277-282. doi:10.1042/CS19990217. PubMed: 10677385. 10677385

[6] Parmentier JH, Muthalif MM, Nishimoto AT, Malik KU (2001) 20-Hydroxyeicosatetraenoic acid mediates angiotensin ii-induced phospholipase d activation in vascular smooth muscle cells. Hypertension 37: 623-629. doi:10.1161/01.HYP.37.2.623. PubMed: 11230346. 11230346

[7] Roman RJ (2002) P-450 metabolites of arachidonic acid in the control of cardiovascular function. Physiol Rev 82: 131-185. PubMed: 11773611. 11773611 10.1152/physrev.00021.2001

[8] Imig JD, Deichmann PC (1997) Afferent arteriolar responses to ANG II involve activation of PLA2 and modulation by lipoxygenase and P-450 pathways. Am J Physiol 273: F274-F282. PubMed: 9277588. 9277588 10.1152/ajprenal.1997.273.2.F274

[9] Dunn KM, Renic M, Flasch AK, Harder DR, Falck J et al. (2008) Elevated production of 20-HETE in the cerebral vasculature contributes to severity of ischemic stroke and oxidative stress in spontaneously hypertensive rats. Am J Physiol Heart Circ Physiol 295: H2455-H2465. doi:10.1152/ajpheart.00512.2008. PubMed: 18952718. 18952718 PMC2614536

[10] Harder DR, Narayanan J, Birks EK, Liard JF, Imig JD et al. (1996) Identification of a putative microvascular oxygen sensor. Circ Res 79: 54-61. doi:10.1161/01.RES.79.1.54. PubMed: 8925569. 8925569

[11] Lübbers DW, Baumgärtl H (1997) Heterogeneities and profiles of oxygen pressure in brain and kidney as examples of the pO_2_ distribution in the living tissue. Kidney Int 51: 372-380. doi:10.1038/ki.1997.49. PubMed: 9027709. 9027709

[12] Ito O, Roman RJ (1999) Regulation of P-450 4A activity in the glomerulus of the rat. Am J Physiol 276: R1749-R1757. PubMed: 10362756. 10362756 10.1152/ajpregu.1999.276.6.R1749

[13] Imig JD, Zou AP, Stec DE, Harder DR, Falck JR et al. (1996) Formation and actions of 20-hydroxyeicosatetraenoic acid in rat renal arterioles. Am J Physiol 270: R217-R227. PubMed: 8769805. 8769805 10.1152/ajpregu.1996.270.1.R217

[14] Guo DF, Inagami T (1994) The genomic organization of the rat angiotensin II receptor AT1B. Biochim Biophys Acta 1218: 91-94. doi:10.1016/0167-4781(94)90105-8. PubMed: 8193170. 8193170

[15] Koike G, Horiuchi M, Yamada T, Szpirer C, Jacob HJ et al. (1994) Human type 2 angiotensin II receptor gene: cloned, mapped to the X chromosome, and its mRNA is expressed in the human lung. Biochem Biophys Res Commun 203: 1842-1850. doi:10.1006/bbrc.1994.2402. PubMed: 7945336. 7945336

[16] Juncos LA, Ito S, Carretero OA, Garvin JL (1994) Removal of endothelium-dependent relaxation by antibody and complement in afferent arterioles. Hypertension 23: I54-I59. doi:10.1161/01.HYP.23.1_Suppl.I54. PubMed: 8282376. 8282376

[17] Ma YH, Gebremedhin D, Schwartzman ML, Falck JR, Clark JE et al. (1993) 20-Hydroxyeicosatetraenoic acid is an endogenous vasoconstrictor of canine renal arcuate arteries. Circ Res 72: 126-136. doi:10.1161/01.RES.72.1.126. PubMed: 8417836. 8417836

[18] Alonso-Galicia M, Hudetz AG, Shen H, Harder DR, Roman RJ (1999) Contribution of 20-HETE to vasodilator actions of nitric oxide in the cerebral microcirculation. Stroke 30: 2727-2734; discussion: 10.1161/01.STR.30.12.2727. PubMed: 10583004. 10583004

[19] Sun CW, Falck JR, Harder DR, Roman RJ (1999) Role of tyrosine kinase and PKC in the vasoconstrictor response to 20-HETE in renal arterioles. Hypertension 33: 414-418. doi:10.1161/01.HYP.33.1.414. PubMed: 9931139. 9931139

[20] Alonso-Galicia M, Falck JR, Reddy KM, Roman RJ (1999) 20-HETE agonists and antagonists in the renal circulation. Am J Physiol 277: F790-F796. PubMed: 10564244. 10564244 10.1152/ajprenal.1999.277.5.F790

[21] Zanetta L, Marcus SG, Vasile J, Dobryansky M, Cohen H et al. (2000) Expression of Von Willebrand factor, an endothelial cell marker, is up-regulated by angiogenesis factors: a potential method for objective assessment of tumor angiogenesis. Int J Cancer 85: 281-288. doi:10.1002/(SICI)1097-0215(20000115)85:2. PubMed: 10629090. 10629090

[22] Horvath B, Hegedus D, Szapary L, Marton Z, Alexy T et al. (2004) Measurement of von Willebrand factor as the marker of endothelial dysfunction in vascular diseases. Exp Clin Cardiol 9: 31-34. PubMed: 19641694. 19641694 PMC2716260

[23] Akishita M, Ito M, Lehtonen JY, Daviet L, Dzau VJ et al. (1999) Expression of the AT2 receptor developmentally programs extracellular signal-regulated kinase activity and influences fetal vascular growth. J Clin Invest 103: 63-71. doi:10.1172/JCI5182. PubMed: 9884335. 9884335 PMC407869

[24] Kim S, Iwao H (2000) Molecular and cellular mechanisms of angiotensin II-mediated cardiovascular and renal diseases. Pharmacol Rev 52: 11-34. PubMed: 10699153. 10699153

[25] Mifune M, Sasamura H, Shimizu-Hirota R, Miyazaki H, Saruta T (2000) Angiotensin II type 2 receptors stimulate collagen synthesis in cultured vascular smooth muscle cells. Hypertension 36: 845-850. doi:10.1161/01.HYP.36.5.845. PubMed: 11082154. 11082154

[26] Zou AP, Ma YH, Sui ZH, Ortiz de Montellano PR, Clark JE et al. (1994) Effects of 17-octadecynoic acid, a suicide-substrate inhibitor of cytochrome P450 fatty acid omega-hydroxylase, on renal function in rats. J Pharmacol Exp Ther 268: 474-481. PubMed: 8301590. 8301590

[27] Ortiz de Montellano PR, Reich NO (1984) Specific inactivation of hepatic fatty acid hydroxylases by acetylenic fatty acids. J Biol Chem 259: 4136-4141. PubMed: 6706995. 6706995

[28] Miyata N, Taniguchi K, Seki T, Ishimoto T, Sato-Watanabe M et al. (2001) HET0016, a potent and selective inhibitor of 20-HETE synthesizing enzyme. Br J Pharmacol 133: 325-329. doi:10.1038/sj.bjp.0704101. PubMed: 11375247. 11375247 PMC1572803

[29] Sadoshima J (1998) Versatility of the angiotensin II type 1 receptor. Circ Res 82: 1352-1355. doi:10.1161/01.RES.82.12.1352. PubMed: 9648733. 9648733

[30] Roman RJ, Alonso-Galicia M (1999) P-450 Eicosanoids: A Novel Signaling Pathway Regulating. Renal Function - News Physiol Sci 14: 238-242. 11390858 10.1152/physiologyonline.1999.14.6.238

[31] Zou AP, Fleming JT, Falck JR, Jacobs ER, Gebremedhin D et al. (1996) 20-HETE is an endogenous inhibitor of the large-conductance Ca(2+)-activated K+ channel in renal arterioles. Am J Physiol 270: R228-R237. PubMed: 8769806. 8769806 10.1152/ajpregu.1996.270.1.R228

[32] Kaley G (2000) Regulation of vascular tone: role of 20-HETE in the modulation of myogenic reactivity. Circ Res 87: 4-5. doi:10.1161/01.RES.87.1.4. PubMed: 10884363. 10884363

[33] Boulay G, Servant G, Luong TT, Escher E, Guillemette G (1992) Modulation of angiotensin II binding affinity by allosteric interaction of polyvinyl sulfate with an intracellular domain of the DuP-753-sensitive angiotensin II receptor of bovine adrenal glomerulosa. Mol Pharmacol 41: 809-815. PubMed: 1569928. 1569928

[34] Imig JD (2013) Epoxyeicosatrienoic acids, 20-hydroxyeicosatetraenoic acid, and renal microvascular function. Prostaglandins Other Lipid Mediat, 104-105: 2–7. PubMed: 23333581. 23333581 10.1016/j.prostaglandins.2013.01.002 PMC3664103

[35] Roman RJ, Maier KG, Sun CW, Harder DR, Alonso-Galicia M (2000) Renal and cardiovascular actions of 20-hydroxyeicosatetraenoic acid and epoxyeicosatrienoic acids. Clin Exp Pharmacol Physiol 27: 855-865. doi:10.1046/j.1440-1681.2000.03349.x. PubMed: 11071299. 11071299

[36] Harder DR, Lange AR, Gebremedhin D, Birks EK, Roman RJ (1997) Cytochrome P450 metabolites of arachidonic acid as intracellular signaling molecules in vascular tissue. J Vasc Res 34: 237-243. doi:10.1159/000159228. PubMed: 9226306. 9226306

[37] Fellner SK, Arendshorst WJ (2005) Angiotensin II Ca2+ signaling in rat afferent arterioles: stimulation of cyclic ADP ribose and IP3 pathways. Am J Physiol Renal Physiol 288: F785-F791. PubMed: 15598842. 15598842 10.1152/ajprenal.00372.2004

[38] Inscho EW, Imig JD, Cook AK (1997) Afferent and efferent arteriolar vasoconstriction to angiotensin II and norepinephrine involves release of Ca2+ from intracellular stores. Hypertension 29: 222-227. doi:10.1161/01.HYP.29.1.222. PubMed: 9039106. 9039106

[39] Jackson WF (2000) Ion channels and vascular tone. Hypertension 35: 173-178. doi:10.1161/01.HYP.35.1.173. PubMed: 10642294. 10642294 PMC1382026

[40] Nowicki S, Chen SL, Aizman O, Cheng XJ, Li D et al. (1997) 20-Hydroxyeicosa-tetraenoic acid (20 HETE) activates protein kinase C. Role in regulation of rat renal Na+,K+-ATPase. J Clin Invest 99: 1224-1230. doi:10.1172/JCI119279. PubMed: 9077530. 9077530 PMC507936

[41] Amlal H, LeGoff C, Vernimmen C, Soleimani M, Paillard M et al. (1998) ANG II controls Na(+)-K+(NH4+)-2Cl- cotransport via 20-HETE and PKC in medullary thick ascending limb. Am J Physiol 274: C1047-C1056. PubMed: 9575802. 9575802 10.1152/ajpcell.1998.274.4.C1047

[42] Cheng J, Wu CC, Gotlinger KH, Zhang F, Falck JR et al. (2010) 20-hydroxy-5,8,11,14-eicosatetraenoic acid mediates endothelial dysfunction via IkappaB kinase-dependent endothelial nitric-oxide synthase uncoupling. J Pharmacol Exp Ther 332: 57-65. doi:10.1124/jpet.109.159863. PubMed: 19841472. 19841472 PMC2802478

[43] Randriamboavonjy V, Busse R, Fleming I (2003) 20-HETE-induced contraction of small coronary arteries depends on the activation of Rho-kinase. Hypertension 41: 801-806. doi:10.1161/01.HYP.0000047240.33861.6B. PubMed: 12623999. 12623999

